# Modified interactive Q-learning for attenuating the impact of model misspecification with treatment effect heterogeneity

**DOI:** 10.1177/09622802231206471

**Published:** 2023-10-20

**Authors:** Yuan Zhang, David M Vock, Megan E Patrick, Thomas A Murray

**Affiliations:** 1 Department of Biostatistics, Epidemiology and Informatics, University of Pennsylvania, Philadelphia, PA, USA; 2 Division of Biostatistics, School of Public Health, University of Minnesota, Minneapolis, MN, USA; 3 Institute for Social Research, 1259 University of Michigan, Ann Arbor, MI, USA

**Keywords:** Dynamic treatment regime, heterogeneous treatment effect, main effect model misspecification, omitted variable bias, sequential multiple assignment randomized trial

## Abstract

A sequential multiple assignment randomized trial, which incorporates multiple stages of randomization, is a popular approach for collecting data to inform personalized and adaptive treatments. There is an extensive literature on statistical methods to analyze data collected in sequential multiple assignment randomized trials and estimate the optimal dynamic treatment regime. Q-learning with linear regression is widely used for this purpose due to its ease of implementation. However, model misspecification is a common problem with this approach, and little attention has been given to the impact of model misspecification when treatment effects are heterogeneous across subjects. This article describes the integrative impact of two possible types of model misspecification related to treatment effect heterogeneity: omitted early-stage treatment effects in late-stage main effect model, and violated linearity assumption between pseudo-outcomes and predictors despite non-linearity arising from the optimization operation. The proposed method, aiming to deal with both types of misspecification concomitantly, builds interactive models into modified parametric Q-learning with Murphy’s regret function. Simulations show that the proposed method is robust to both sources of model misspecification. The proposed method is applied to a two-stage sequential multiple assignment randomized trial with embedded tailoring aimed at reducing binge drinking in first-year college students.

## 1. Introduction

A sequential multiple assignment randomized trial (SMART)^1,2^ is a trial in which some or all participants undergo multiple stages of randomization. The data collected in such trials can be used by investigators to construct and improve dynamic treatment regimes (DTRs).^3,4^ A DTR is a sequence of decision-making functions, one at each stage, which map from a subject’s history of characteristics, interventions, and responses to previous interventions to a recommended intervention. Making treatment decisions dynamically based on evolving patient information has become an important clinical practice that takes treatment effect heterogeneity into account and effectuates personalized medicine.

Q-learning with linear regression^5,6^ is a widely used backward induction algorithm^7^ to identify the optimal DTR. For each stage of treatment, Q-learning requires the correct specification of a Q-function,^3^ which is a parametric model for the expected outcome conditional on past history while assuming that the optimal interventions are followed thereafter. To ease implementation and interpretation, the Q-function is commonly modeled using linear regression, with a main effect component and a treatment effect component. The main effect component characterizes variation in the outcome that can be explained by pre-treatment covariates, whereas the treatment effect component characterizes the average effect of the observed/assigned treatment at the relevant stage allowing for variation with pre-treatment covariates.

Decision making at a single stage, for example, using data from a randomized controlled trial, does not depend on the main effect model as the treatment effect model fully defines the estimated optimal rule. This is not the case for a backward induction process over multiple stages like Q-learning. The standard Q-learning algorithm is susceptible to model misspecification of the main effect component of the Q-function. Heterogeneous treatment effects at an earlier stage on a final outcome are in fact part of the main effects at later stages (either as the earlier treatment-covariate interaction or as an intermediate measurement which depends on prior treatment). In practice, early-stage treatment interactions are usually omitted in late-stage main effect model, probably out of consideration for interpretability, overfitting and convergence issues, and doing so will result in biased estimation of the treatment effects at early stages. This is an example of (informative) residual bias in optimizing over multiple stages and is problematic in identifying the optimal DTR. Existing methods that deal with residual bias are modified Q-learning,^8^ A-learning,^9^ and robust Q-learning.^10^ A-learning takes a propensity score approach and allows for flexible modeling of the main effects. Robust Q-learning as well takes a propensity score approach, but obviates the need to specify the main effect model. Nonparametric methods are usually used to estimate the main effects in A-learning and the expected outcome in robust Q-learning. Nonparametric methods work ideally for nonlinearity between outcomes and covariates, but are less straightforward to interpret and implement. Moreover, model checking and residual diagnostics for Q-learning with linear regression can be easily performed using standard approaches.^11,12^ Therefore, we advocate the use of modified Q-learning, a parametric approach that takes account of stage 2 residuals, for dealing with misspecification of the main effect model.

Additionally, a nonzero treatment effect at a later stage results in biased estimation of early-stage linear models.^13^ Although the treatment effect component of a Q-function is assumed to be correctly specified with no unmeasured confounders, a nonlinear relationship between early-stage pseudo-outcome and predictors arises from the optimization operation when the late-stage treatment effects are nonzero. Therefore, late-stage heterogeneous treatment effects, if present, may bias the estimation of early-stage Q-function because the treatment effects are highly likely to be nonzero across all patients, especially when the associated patient characteristic is on a continuous scale. To deal with violation of the linearity assumption, Laber et al.^13^ proposed an interactive model building of Q-learning to correct the bias caused by the misspecified linearity between pseudo-outcome and predictors.

For a two-stage SMART, the two types of misspecification described above are associated with heterogeneous treatment effects at stage 1 and stage 2, respectively. Both of them result in a nonnegligible bias in the prediction of stage 1 optimal rule using Q-learning and have been addressed individually with the carefully constructed methods as discussed earlier. However, investigators of many SMART studies would expect heterogeneous treatment effects at both stages and thus these two sources of model misspecification need to be addressed together. Our motivating example is the M-bridge study,^14,15^ a two-stage SMART that develops and evaluates the DTRs to reduce binge drinking and related consequences among first-year college students. The investigators recorded at baseline a comprehensive set of covariates, including demographics, pre-college drinking norms, intention for college drinking, and pre-college drinking habits, which would potentially moderate the intervention effect at both stages. In a standard analysis using Q-learning,^16^ we typically are reluctant to include heterogeneous treatment effects from earlier stages in the current-stage Q-function because, first, inclusion of all interactions between baseline covariates and earlier treatments may cause overfitting problems, and second, Q-learning works backwards and we cannot have identified important interactions from earlier stages to include in the current-stage estimation. If we have multiple stages, then the overfitting problem would accumulate because the Q-function at the k th stage should include k -way interactions in the main effect model to avoid any potential residual bias. We focus on the two-stage setting (as observed in the M-bridge study) in this article, in which omission of important stage 1 heterogeneous treatment effects in the stage 2 Q-function would cause residual bias, and the linearity of stage 1 Q-function is highly likely misspecified due to stage 2 heterogeneous treatment effects. Therefore, there is a strong need for considering the coexistence of the abovementioned misspecifications and bridging the methods to deal with both types of misspecification simultaneously.

We begin with an introduction of the data structure and a further elaboration on the importance of the problem using the M-bridge study as an example in Section 2. We then discuss the integrative impact of late-stage unadjusted residuals and early-stage nonlinearity on the prediction of optimal rules, with mathematical formulation in Section 3 to help understand the statistical aspects of the problem. Specifically, we outline the proof of residual bias in the Supplemental Materials to fill the gaps in the DTR literature. We then propose to build interactive models into modified parametric Q-learning with Murphy’s regret function in Section 4. Simulations are performed in Section 5 to show the robustness of our proposed algorithm with heterogeneous treatment effects at both stages. We then demonstrate its application on SMARTs with embedded tailoring using the M-bridge data in Section 6. Finally, we conclude with a discussion in Section 7.

## 2. Background

### 2.1. Data and framework

**Figure 1.**
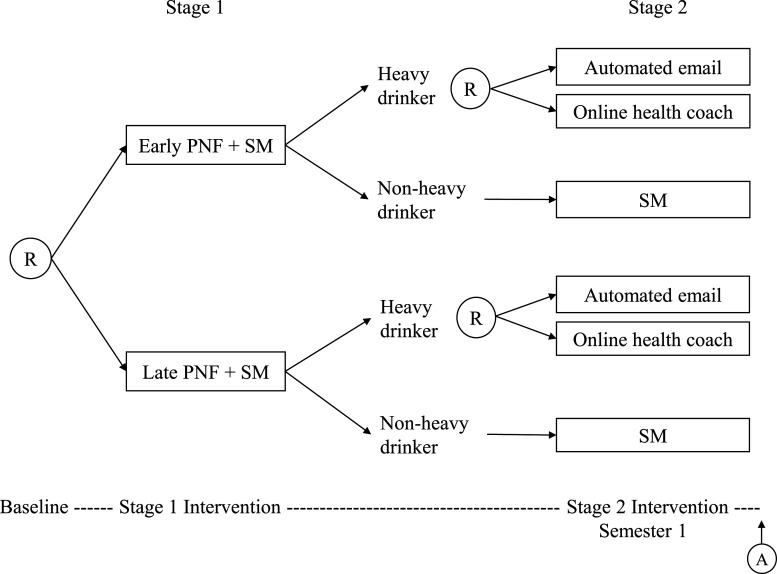
The M-bridge study: a sequential, multiple assignment, randomized trial. This figure is adapted from the figure of study design by Patrick et al.^14^ Ⓡ indicates a randomization stage with arrows pointing to available treatment options, and Ⓐ indicates an assessment of outcomes. Note that this design only considers a final outcome measured at the end of the treatment course.

We use the M-bridge study as a data example to illustrate the problem. As shown in Figure 1, the M-bridge study has two stages of intervention. At stage 1, enrolled students were randomized to receive a combined universal preventive intervention consisting of personalized normative feedback and self-monitoring (PNF+SM), either starting prior to attending college (early intervention) or in the first month of Semester 1 (late intervention). Two intermediate measures of alcohol use, namely, the frequency of binge drinking (consuming 4/5+ drinks in a row for women/men) and the frequency of high-intensity drinking (consuming 8/10+ drinks in a row for women/men) in the past two weeks, were reported by students in four self-monitoring surveys. Students were flagged as a “heavy drinker” if they reported at any self-monitoring survey two or more occasions of binge drinking, or one or more occasion of high-intensity drinking. The self-monitoring period ended once a student was flagged as a heavy drinker. At stage 2, heavy drinkers were re-randomized to either an automated email or an invitation to online health coaching so as to bridge these eligible students to indicated interventions, whereas non-heavy drinkers continued self-monitoring for the rest of Semester 1. Binge drinking (primary outcome) and negative drinking-related consequences (secondary outcome) were measured at the end of Semester 1 for all students.

Our scientific question is to identify the DTR that minimizes the maximum number of drinks consumed within a 24-hour period, and the total number of alcohol-related consequences in the past 30 days, which was measured using 24 items from the Brief Young Adult Alcohol Consequences Questionnaire (B-YAACQ), respectively. In other words, our aim is (1) to determine whether a student should receive PNF+SM early or late, conditional on their baseline characteristics, and (2) whether a student should receive an automated email or online health coaching if they are a heavy drinker but continue self-monitoring if they are not a heavy drinker, so that the combined regime minimizes problematic drinking. In the M-bridge study, the investigators hypothesize that pre-college alcohol use norms and pre-college intentions for college drinking are the treatment effect moderators at stage 1, and intermediate binge drinking is the treatment effect moderator at stage 2. We also evaluate effect moderation with respect to demographics and pre-college drinking habits.

We assume a two-stage setting throughout the paper, although the following could be generalized to multiple decision points. Suppose that the data collected from a SMART are represented by a sequence of independent and identically distributed random variables $\left(Z_{1},A_{1},Z_{2},A_{2},Y\right)$ , where $Z_{1}$ is the set of baseline covariates and potential moderators measured prior to stage 1 randomization, $Z_{2}$ is the set of time-varying covariates and tailoring variables measured after stage 1 and before stage 2 randomization, $A_{k}\in A_{k}$ , k=1,2 , is the treatment that the participant receives at stage k , with $A_{k}$ being the set of all possible treatments, and Y is the outcome measured after treatment stages, with smaller values preferred.

In the M-bridge study, $A_{1}=\{-1,1\}$ , where $A_{1}=1$ represents the early intervention and $A_{1}=-1$ represents the late intervention, and $A_{2}=\{-1,1\}$ , where $A_{2}=1$ represents an online health coach and $A_{2}=-1$ represents an automated email. $Z_{1}$ includes the baseline information on subject characteristics (race, gender, and intention to pledge to a sorority or fraternity [i.e. “Greek”], indicator whether parents have a significant drinking problem), pre-college norms and intention for college drinking, and pre-college drinking habits. $Z_{2}$ includes solely the embedded tailoring variable, that is, the flag of heavy drinkers. Y can be either the maximum number of drinks consumed within a 24-hour period or the total number of negative alcohol-related consequences in the past 30 days. Let $H_{1}=Z_{1}$ and $H_{2}=(Z_{1},A_{1},Z_{2})$ denote the covariate and treatment history up to the stage 1 and stage 2 randomization, respectively.

Optimization of DTRs relies on the concept of potential outcomes, and therefore the following causal assumptions are necessary: (i) consistency: the potential outcome under the observed treatment agrees with the observed outcome; (ii) no unmeasured confounders, also known as sequential ignorability: $A_{k}$ is an independent of all future potential outcomes, conditional on the history $H_{k}$ ; and (iii) positivity: $P(A_{k}=a_{k}|H_{k})>0$ for all $a_{k}\in A_{k}$ . The no unmeasured confounders assumption is valid with the sequential randomization used in a SMART. In addition to the positivity assumption, we further assume that the randomization scheme has a fixed allocation ratio with $A_{k}$ specified such that $E(A_{k})=0$ . A detailed discussion of this assumption can be found in Section 7.

### 2.2. Q-learning

We describe the algorithm of standard Q-learning with linear regression to identify the optimal DTR. Starting from stage 2, the Q-function is specified as

$$Q_{2}(H_{2},A_{2})=E(Y|H_{2},A_{2})=\beta_{200}+X_{20}^{T}\beta_{201}+A_{2}\left(\beta_{210}+X_{21}^{T}\beta_{211}\right)$$

where $X_{20}$ and $X_{21}$ denote the vectors formed by elements in $H_{2}$ that represent the predictors in stage 2 main effect model and treatment effect model, respectively. The estimators of the parameters, ${\hat{\beta }}_{200},\;{\hat{\beta }}_{201},\;{\hat{\beta }}_{210},\mathrm{and}\;\;{\hat{\beta }}_{211}$ , are obtained using ordinary least squares. For the preceding stage (i.e. stage 1), the Q-function is specified as

$$\begin{array}{rl} Q_{1}(H_{1},A_{1}) & =E\left\{\min_{a_{2}\in A_{2}}Q_{2}(H_{2},A_{2}=a_{2})|H_{1},A_{1}\right\}\\ & =\beta_{100}+X_{10}^{T}\beta_{101}+A_{1}\left(\beta_{110}+X_{11}^{T}\beta_{111}\right) \end{array}$$

where $X_{10}$ and $X_{11}$ denote the vectors formed by elements in $H_{1}$ that represent the predictors in stage 1 main effect model and treatment effect model, respectively. The estimators of the parameters, ${\hat{\beta }}_{100},\;{\hat{\beta }}_{101},\;{\hat{\beta }}_{110},\mathrm{and}\;\;{\hat{\beta }}_{111}$ , are obtained using ordinary least squares. The predicted optimal DTR is $\left({\hat{d}}_{1}^{\text{opt}},\;{\hat{d}}_{2}^{\text{opt}}\right)$ , with

$${\hat{d}}_{j}^{\text{opt}}=\underset{a_{j}\in A_{j}}{\arg\;\min}\;Q_{j}(H_{j},A_{j}=a_{j};{\hat{\beta }}_{j00},{\hat{\beta }}_{j01},{\hat{\beta }}_{j10},{\hat{\beta }}_{j11})\;\text{ for }j=1,2$$

## 3. Misspecification with treatment effect heterogeneity

In this section, the integrated impact of the two sources of model misspecification caused by heterogeneous treatment effects are formulated mathematically and discussed in detail. We use uppercase to denote random variables and lowercase to denote a realization of the corresponding random variable. Suppose Y follows an independent and identical distribution with conditional expectation given by

(1) $$E(Y|H_{2},A_{2})=\psi_{200}+x_{20}^{T}\psi_{201}+a_{1}x_{11}^{T}\gamma_{20}+a_{2}\left(\psi_{210}+x_{21}^{T}\psi_{211}\right)$$

where $\psi_{200}$ , $\psi_{201}$ , $\psi_{210}$ , $\psi_{211}$ , and $\gamma_{20}$ denote the true parameters, and $X_{20}=X_{21}=(X_{10}^{T},\;A_{1},\;Z_{2}{)}^{T}$ does not include any important interaction between baseline covariates and stage 1 treatment $A_{1}X_{11}$ . Therefore, $a_{1}x_{11}^{T}\gamma_{20}$ represents the stage 1 heterogeneous treatment effect and $a_{2}x_{21}^{T}\psi_{211}$ represents the stage 2 heterogeneous treatment effect. We assume that $A_{1}$ and $A_{2}$ are specified such that $E(A_{1})=E(A_{2})=0$ .

In the backward induction setting, stage 1 optimization is contingent on compliance with the optimal rule at stage 2, so the true optimal pseudo-outcome at stage 1 is

$$Y^{\text{opt}}=E\left(Y|H_{2},A_{2}=d_{2}^{\text{opt}}\right)=\psi_{200}+x_{20}^{T}\psi_{201}+a_{1}x_{11}^{T}\gamma_{20}-|\psi_{210}+x_{21}^{T}\psi_{211}|$$

where $d_{2}^{\text{opt}}$ is the optimal decision rule at stage 2 and

$$d_{2}^{\text{opt}}(H_{2})=-\text{sgn}\left\{\psi_{210}+x_{21}^{T}\psi_{211}\right\}$$

with sgn(x)=−1 if x<0 and 1 otherwise.

### 3.1. Omitted stage 1 heterogeneous treatment effects in the stage 2 model

Omitted stage 1 heterogeneous treatment effects in the stage 2 main effect model may cause a loss in the power to correctly predict stage 1 optimal rules. Investigators may miss the importance of properly adjusting for stage 1 treatment effects in the stage 2 model as only the (heterogeneous) treatment effects of stage 2 intervention impact the stage 2 optimal rule. In fact, Q-learning with linear regression is often implemented using a linear predictor function such that the same design matrix is used for both the main effect and treatment effect models. Moreover, three-way interactions are rarely included in the stage 2 treatment model, so $X_{21}$ often does not include interactions between $A_{1}$ and baseline covariates. However, doing so may result in a bias in stage 1 estimation. Furthermore, investigators should be alert to this issue in the use of Q-learning software. qLearn^17^ allows for different and explicit specifications of the main effect and treatment effect model, but it is less straightforward in qlaci^18^ and iqLearn.^19^

To understand the problem thoroughly, we provide a full argument of residual bias based on the omitted variable bias theorem in Supplemental Appendix A. Equation (1) is a special case of equation (A.1). Suppose the stage 2 Q-function, $Q_{2}(H_{2},A_{2})=\beta_{200}+X_{20}^{T}\beta_{201}+A_{2}(\beta_{210}+X_{21}^{T}\beta_{211})$ , omits $A_{1}X_{11}$ . Substituting the stage 2 predictors $X_{2}=(X_{20}^{T},\;A_{2},\;A_{2}X_{21}^{T}{)}^{T}$ and the unmeasured variables $V_{20}=A_{1}X_{11}$ in equation (A.2), the bias of stage 2 main effect estimators is

(2) $$B_{s}^{\prime }=\left(\begin{array}{c} E(A_{1}X_{11}^{T})-E(X_{2}^{T})\text{Cov}(X_{2}{)}^{-1}\text{Cov}(X_{2},A_{1}X_{11})\\ \text{Cov}(X_{20}{)}^{-1}\left(\begin{array}{c} E(A_{1})\text{Cov}(X_{10},X_{11})\\ \text{Var}(A_{1})E(X_{11}^{T})\\ \text{Cov}(Z_{2},A_{1}X_{11}) \end{array}\right) \end{array}\right)$$

Even if $E(A_{1})=0$ , $B_{s}^{\prime }$ is a nonzero vector. Therefore, wrongly omitting stage 1 heterogeneous treatment effects results in biased estimation of stage 2 main effects.

### 3.2. Nonlinearity of stage 1 pseudo-outcome

To simplify subsequent notation, we define ${\tilde{X}}_{20}=(1,\;X_{20}^{T}{)}^{T}$ , ${\tilde{X}}_{21}=(1,\;X_{21}^{T}{)}^{T}$ , $\psi_{20}=(\psi_{200},\;\psi_{201}^{T}{)}^{T}$ , $\psi_{21}=(\psi_{210},\;\psi_{211}^{T}{)}^{T}$ , and rewrite $Y^{\text{opt}}$ as $Y^{\text{opt}}={\tilde{x}}_{20}^{T}\psi_{20}+a_{1}x_{11}^{T}\gamma_{20}-|{\tilde{x}}_{21}^{T}\psi_{21}|$ . The stage 2 optimal treatment effect, $-|{\tilde{X}}_{21}^{T}\psi_{21}|$ , is a non-smooth function in $\psi_{21}$ . Nonregularity of stage 1 parameters due to the non-smooth function $-|{\tilde{X}}_{21}^{T}\psi_{21}|$ has been extensively studied in literature.^20,21^ In order to satisfy the regularity conditions for statistical inference, we assume

(3) $$P\{H_{2}:{\tilde{X}}_{21}^{T}\psi_{21}=0\}=0$$

The estimator of $Y^{\text{opt}}$ is

$${\hat{Y}}^{\text{opt}}=\min_{a_{2}}Q_{2}({\tilde{x}}_{2},a_{2};{\hat{\beta }}_{200},{\hat{\beta }}_{201},{\hat{\beta }}_{210},{\hat{\beta }}_{211})={\tilde{x}}_{20}^{T}{\hat{\beta }}_{20}-|{\tilde{x}}_{21}^{T}{\hat{\beta }}_{21}|$$

where ${\hat{\beta }}_{20}=({\hat{\beta }}_{200},\;{\hat{\beta }}_{201}^{T}{)}^{T}$ and ${\hat{\beta }}_{21}=({\hat{\beta }}_{210},\;{\hat{\beta }}_{211}^{T}{)}^{T}$ . For large samples, ${\hat{\beta }}_{21}$ is a consistent estimator of $\psi_{21}$ . Under Assumption (3), $|{\tilde{x}}_{21}^{T}{\hat{\beta }}_{21}|$ is also a consistent estimator of $|{\tilde{x}}_{21}^{T}\psi_{21}|$ by the continuous mapping theorem. However, $|{\tilde{x}}_{21}^{T}{\hat{\beta }}_{21}|$ is a biased estimator of $|{\tilde{x}}_{21}^{T}\psi_{21}|$ as $|{\tilde{x}}_{21}^{T}\psi_{21}|\neq 0$ . Normality is usually assumed for the conditional distribution of stage 2 effects on stage 1 covariates, but in stage 1 estimation, bias can still be induced by the absolute value function $|{\tilde{x}}_{21}^{T}\psi_{21}|$ . Q-learning requires the causal assumption of no unmeasured confounders to be satisfied in order to obtain unbiased estimators of treatment effects, that is, the treatment effect model at each stage is correctly specified. Thus, the bias discussed here does not result from misspecification of the treatment effect model, but intrinsically from the misspecified linear relationship between the pseudo-outcome and stage 1 covariates when stage 2 treatment effect is nonzero and heterogeneous, as a result of the optimization operation and the absolute value function. The detailed proof of the nonlinear relationship can be found in the Supplemental Materials of Laber et al.^13^

### 3.3. Integrative impact of model misspecification

Now we derive an expression for stage 1 bias from model misspecification associated with heterogeneous treatment effects at both stages. First, we rewrite the stage 1 Q-function as $Q_{1}(H_{1},A_{1})=\beta_{100}+X_{10}^{T}\beta_{101}+a_{1}(\beta_{110}+X_{11}^{T}\beta_{111})\equiv {\tilde{X}}_{10}^{T}\beta_{10}+a_{1}{\tilde{X}}_{11}^{T}\beta_{11}$ , where ${\tilde{X}}_{10}=(1,\;X_{10}^{T}{)}^{T}$ , ${\tilde{X}}_{11}=(1,\;X_{11}^{T}{)}^{T}$ , $\beta_{10}=(\beta_{100},\;\beta_{101}^{T}{)}^{T}$ , and $\beta_{11}=(\beta_{110},\;\beta_{111}^{T}{)}^{T}$ . The values of the stage 1 parameters can be obtained as

$$(\beta_{10},\beta_{11})=\underset{\beta_{10},\beta_{11}}{\arg\;\min}\;E\left[{\left\{{\tilde{X}}_{20}^{T}\beta_{20}-|{\tilde{X}}_{21}^{T}\beta_{21}|-{\tilde{X}}_{10}^{T}\beta_{10}-A_{1}{\tilde{X}}_{11}^{T}\beta_{11}\right\}}^{2}\right]$$

Let ${\hat{\beta }}_{10}$ and ${\hat{\beta }}_{11}$ be the corresponding ordinary least squares estimators. Suppose ${\tilde{X}}_{1}=({\tilde{X}}_{10}^{T},\;A_{1}{\tilde{X}}_{11}^{T}{)}^{T}$ is the vector of predictors and ${\tilde{\text{X}}}_{1}=({\tilde{\text{X}}}_{10},\;A_{1}{\tilde{\text{X}}}_{11})$ is the design matrix for the stage 1 estimation and is of full column rank, and $Y^{\text{opt}}$ is the outcome vector with $Y_{i}^{\text{opt}}$ as the i th element, i=1,…,n . Then the estimators of stage 1 model parameters are

$$\begin{array}{rl} \left(\begin{array}{c} {\hat{\beta }}_{10}\\ {\hat{\beta }}_{11} \end{array}\right) & =({\tilde{\text{X}}}_{1}^{T}{\tilde{\text{X}}}_{1}{)}^{-1}{\tilde{\text{X}}}_{1}^{T}{\hat{Y}}^{\text{opt}}\\ & =({\tilde{\text{X}}}_{1}^{T}{\tilde{\text{X}}}_{1}{)}^{-1}{\tilde{\text{X}}}_{1}^{T}({\tilde{\text{X}}}_{20}{\hat{\beta }}_{20}-|{\tilde{\text{X}}}_{21}{\hat{\beta }}_{21}|) \end{array}$$

and the bias of stage 1 estimation is

(4) $$\begin{array}{rl} E({\tilde{x}}_{10}^{T}{\hat{\beta }}_{10}+a_{1}{\tilde{x}}_{11}^{T}{\hat{\beta }}_{11})-E\left(Y^{\text{opt}}|{\tilde{x}}_{1},a_{1}\right) & ={\tilde{x}}_{1}^{T}E\left\{({\tilde{\text{X}}}_{1}^{T}{\tilde{\text{X}}}_{1}{)}^{-1}{\tilde{\text{X}}}_{1}^{T}({\tilde{\text{X}}}_{20}{\hat{\beta }}_{20}-|{\tilde{\text{X}}}_{21}{\hat{\beta }}_{21}|)\right\}\\ & \;-E\left({\tilde{x}}_{20}^{T}\psi_{20}+a_{1}x_{11}^{T}\gamma_{20}-|{\tilde{x}}_{21}^{T}\psi_{21}||H_{1},A_{1}\right)\\ & =\left[{\tilde{x}}_{1}^{T}E\left\{({\tilde{\text{X}}}_{1}^{T}{\tilde{\text{X}}}_{1}{)}^{-1}{\tilde{\text{X}}}_{1}^{T}B_{s}^{\prime }\right\}-a_{1}x_{11}^{T}\right]\gamma_{20} \end{array}$$

(5) $$\begin{array}{rl} & \;+\left[E\left(|{\tilde{x}}_{21}^{T}\psi_{21}||{\tilde{x}}_{1},a_{1}\right)-{\tilde{x}}_{1}^{T}E\left\{({\tilde{\text{X}}}_{1}^{T}{\tilde{\text{X}}}_{1}{)}^{-1}{\tilde{\text{X}}}_{1}^{T}E\left(|{\tilde{\text{X}}}_{21}{\hat{\beta }}_{21}||H_{1},A_{1}\right)\right\}\right] \end{array}$$

where $B_{s}^{\prime }$ is the expression in equation (2). Bias (4) is induced by the omission of stage 1 heterogeneous treatment effects in the stage 2 main effect model, and bias (5) is induced by falsely assuming linearity between the absolute value of stage 2 heterogeneous treatment effects and stage 1 predictors.

## 4. The proposed method

### 4.1. The modified interactive Q-learning algorithm

Interactive Q-learning^13^ was proposed to address the misspecified linearity in stage 1 estimation by separately regressing stage 2 main effects on stage 1 predictors and estimating the conditional distribution of stage 2 treatment effects conditional on stage 1 predictors, and then combining the former and the expected absolute value of the latter to get the estimated stage 1 Q-function. To address both types of bias simultaneously, our proposed method follows the virtue of interactive Q-learning and modifies the main effect portion of the algorithm to account for any informative residuals from stage 2 estimation. The modified interactive Q-learning (mIQ) algorithm comprises the following steps:

(mIQ-1)
Regress Y on $H_{2},A_{2}$ based on the stage 2 Q-function $Q_{2}(H_{2},A_{2};\beta_{20},\beta_{21})={\tilde{X}}_{20}^{T}\beta_{20}+A_{2}{\tilde{X}}_{21}^{T}\beta_{21}$ to obtain the ordinary least squares estimators ${\hat{\beta }}_{20},{\hat{\beta }}_{21}$ ;
(mIQ-2)
Regress $Y-a_{2}{\tilde{x}}_{21}^{T}{\hat{\beta }}_{21}$ on $H_{1},A_{1}$ to obtain the consistent estimator of $E(Y-A_{2}{\tilde{X}}_{21}^{T}\psi_{21}|H_{1},A_{1})$ , denoted by $\hat{m}(H_{1},A_{1})$ ;
(mIQ-3)
Estimate the conditional distribution $g({\tilde{X}}_{21}^{T}\psi_{21}|H_{1},A_{1})$ , denoted by $\hat{g}(\cdot |H_{1},A_{1})$ : If g is a conditional normal density with constant variance, that is, ${\tilde{X}}_{21}^{T}\psi_{21}|H_{1},A_{1}∼N(\mu (H_{1},A_{1}),\sigma^{2})$ , then regress ${\tilde{x}}_{21}^{T}{\hat{\beta }}_{21}$ on $H_{1},A_{1}$ to obtain the estimators $\hat{\mu }(H_{1},A_{1})$ and $\hat{\sigma }$ ;
(mIQ-4)
Obtain the estimator of $E(Y^{\text{opt}}|H_{1},A_{1})=E({\tilde{X}}_{20}^{T}\psi_{20}-|{\tilde{X}}_{21}^{T}\psi_{21}||H_{1},A_{1})$ by combining the above estimators:
$${\hat{Q}}_{1}(H_{1},A_{1})=\hat{m}(H_{1},A_{1})-\int |z|\hat{g}(z|H_{1},A_{1})dz$$
  where the integral can be easily calculated for a location-scale distribution g .

The optimal DTR is identified as $\left({\hat{d}}_{1}^{\text{opt}},\;{\hat{d}}_{2}^{\text{opt}}\right)$ , where ${\hat{d}}_{1}^{\text{opt}}={\arg\;\min}_{a_{1}\in \{-1,1\}}\;{\hat{Q}}_{1}(H_{1}=h_{1},A_{1}=a_{1})$ and ${\hat{d}}_{2}^{\text{opt}}=-\text{sgn}\{{\tilde{x}}_{21}^{T}{\hat{\beta }}_{21}\}$ . The contrast with the interactive Q-learning algorithm is Step **(mIQ-3)**, where this modified algorithm incorporates any stage 2 residual remainder from misspecification of the main effect model. As ${\hat{\beta }}_{21}$ is an consistent estimator of $\psi_{21}$ , $\hat{m}(H_{1},A_{1})$ is a consistent estimator of $E(Y-A_{2}{\tilde{X}}_{21}^{T}\psi_{21}|H_{1},A_{1})$ .

### 4.2. Small sample properties of the proposed estimator

The pseudo-outcome in the stage 1 estimation, $Y^{\text{opt}}=E(Y|H_{2},A_{2}=d_{2}^{\text{opt}}(H_{2}))$ can be a counterfactual outcome. If $d_{2}^{\text{opt}}=a_{2}$ , then the expression represents the expected outcome under the assigned treatment at stage 2; if $d_{2}^{\text{opt}}\neq a_{2}$ , then it represents the expected outcome under the counterfactual treatment. We observe that $Y^{\text{opt}}=E(Y|H_{2},A_{2}=a_{2})-21\{d_{2}^{\text{opt}}\neq a_{2}\}|{\tilde{x}}_{21}^{T}\psi_{21}|=E(Y|H_{2},A_{2}=a_{2})+(d_{2}^{\text{opt}}-a_{2}){\tilde{x}}_{21}^{T}\psi_{21}$ , where $1\{d_{2}^{\text{opt}}\neq a_{2}\}=1$ if $d_{2}^{\text{opt}}\neq a_{2}$ and 0 otherwise. Hence, the estimator of stage 1 Q-function, ${\hat{Q}}_{1}(H_{1},A_{1})$ , has a bias of the form

(6) $$\begin{array}{rl} \text{Bias}({\hat{Q}}_{1}) & =E\left\{{\tilde{X}}_{1}^{T}({\tilde{X}}_{1}{\tilde{X}}_{1}^{T}{)}^{-1}{\tilde{X}}_{1}(Y-A_{2}{\tilde{X}}_{21}^{T}{\hat{\beta }}_{21})\right\}-E\left\{E_{\hat{g}}(|{\tilde{X}}_{21}^{T}\psi_{21}||H_{1},A_{1})\right\}\\ & \;-E\left\{Y+(d_{2}^{\text{opt}}-A_{2}){\tilde{X}}_{21}^{T}\psi_{21}|H_{1},A_{1}\right\}\\ & =E\left\{{\tilde{X}}_{1}^{T}({\tilde{X}}_{1}{\tilde{X}}_{1}^{T}{)}^{-1}{\tilde{X}}_{1}Y\right\}-E(Y|H_{1},A_{1}) \end{array}$$

(7) $$\begin{array}{rl} & -\left[E\left\{E_{\hat{g}}(|{\tilde{X}}_{21}^{T}\psi_{21}||H_{1},A_{1})\right\}-E\left(|{\tilde{X}}_{21}^{T}\psi_{21}||H_{1},A_{1}\right)\right] \end{array}$$

Therefore, unbiased estimation of stage 1 Q-function requires the stage 1 model to be correctly specified so that the linearity between Y and stage 1 predictors is valid (shown by Expression (6)), and the assumption of normality of the underlying distribution g to be true so that estimation of the conditional distribution of stage 2 treatment effects on stage 1 predictors is unbiased (shown by Expression (7)). Laber et al.^13^ proposed additional nonparametric modeling of g using the empirical distribution, which helps to loosen the latter assumption of normality and increase the modeling flexibility of this algorithm.

## 5. Simulation study

We conducted a simulation study to show the *predictive* performance of the proposed method (mIQ) in the context of small samples, and compared mIQ with standard Q-learning (Q), modified Q-learning (mQ),^8^ and interactive Q-learning (IQ).^13^ Two metrics were used to assess these methods: probability of correctly identified (PCI) stage 1 optimal rules, and bias of the estimated optimal value. Biased estimators of treatment effects may lead to incorrect decision making. Though we have discussed extensively the bias of parameter estimators using Q-learning, eventually we care about identifying the correct decision rules. The optimal value,^22^ $E(Y|A_{1}=d_{1}^{\text{opt}},A_{2}=d_{2}^{\text{opt}})$ , is the expected outcome under the optimal DTR and is estimated by ${\hat{Q}}_{1}(H_{1}=h_{1},A_{1}={\hat{d}}_{1}^{\text{opt}})$ . The bias of the estimated optimal value reflects the combined bias in estimation of stage 1 main effects and treatment effects. To empirically verify our argument of omitted variable bias described in Section 3.1 and the theorems stated in Supplemental Appendix A, we conducted a preliminary study to examine the bias of the stage 2 main effect and treatment effect estimators (see Supplemental Appendix C). The preliminary results also show that omission of stage 1 heterogeneous treatment effects in the stage 2 main effect model causes significant bias in identifying the stage 1 optimal rules.

We assumed that a sequence of observations from a SMART study was $\left(Z_{1i},A_{1i},Z_{2i},A_{2i},Y_{i}\right)$ , i=1,…,n , where $Z_{1i}\overset{\text{i.i.d.}}{∼}N(-2,1)$ , $Z_{2i}=Z_{1i}+\phi_{i}$ and $\phi_{i}\overset{\text{i.i.d.}}{∼}N(0,4)$ , $A_{1i}\overset{\text{i.i.d.}}{∼}2\text{Bernoulli}(0.5)-1$ and $A_{2i}\overset{\text{i.i.d.}}{∼}2\text{Bernoulli}(0.5)-1$ . There was no embedded tailoring variable in this design. Suppose $Y_{i}={\tilde{X}}_{20,i}^{T}\psi_{20}+c_{2}A_{2i}{\tilde{X}}_{21,i}^{T}\psi_{21}+\varepsilon_{i}$ , where $\varepsilon_{i}\overset{\text{i.i.d.}}{∼}N(0,1)$ , and small values of $Y_{i}$ were preferred. Note that $c_{2}$ indicates the size and direction of stage 2 heterogeneous treatment effects.

For a data generative process with specifications of ${\tilde{X}}_{20,i}^{T}\psi_{20}$ and ${\tilde{X}}_{21,i}^{T}\psi_{21}$ , we evaluated the estimators by Monte Carlo integration using samples of size n=250 to predict the individualized optimal DTR for a known population, which was represented by a functional equivalent of generating a dataset of N=10,000 subjects with potential outcomes under the four treatment regimes. Hence, the true optimal rules, $d_{1}^{\text{opt}}$ and $d_{2}^{\text{opt}}$ , are known for each subject in the population by minimizing the expected potential outcomes over all treatment regimes. The general framework described in Section 2.1 defines $Z_{2}$ as the set of stage 2 time-varying covariates and tailoring variables. Note that in our simulation setting, $Z_{2}$ does not vary with $A_{1}$ in predicting ${\hat{d}}_{2}^{\text{opt}}$ . Therefore, we do not need to worry about the change in $Z_{2}$ with respect to ${\hat{d}}_{1}^{\text{opt}}$ which in turn affects the prediction of ${\hat{d}}_{2}^{\text{opt}}$ .

**Table 1.** Bias (mean (SD)) of the estimated optimal value using standard Q-learning (Q), modified Q-learning (mQ), interactive Q-learning (IQ), and the proposed method (mIQ), based on a set of the population data ( N=10,000 ) and 1000 simulations of the sample data ( n=250 ).

$c_{1}$	$c_{2}$	Q	mQ	IQ	mIQ
0.0	1.0	0.38 (0.16)	0.38 (0.16)	0.00 (0.11)	0.00 (0.12)
	2.0	0.76 (0.24)	0.76 (0.24)	0.03 (0.12)	0.03 (0.13)
	3.0	1.22 (0.37)	1.22 (0.37)	0.06 (0.14)	0.05 (0.15)
2.0	1.0	0.80 (0.30)	0.28 (0.23)	0.73 (0.28)	0.01 (0.23)
	2.0	1.53 (0.32)	0.70 (0.28)	1.05 (0.27)	0.06 (0.23)
	3.0	2.14 (0.39)	1.16 (0.37)	1.20 (0.28)	0.08 (0.25)
4.0	1.0	0.71 (0.50)	0.23 (0.38)	0.70 (0.50)	0.04 (0.40)
	2.0	1.68 (0.52)	0.60 (0.41)	1.55 (0.50)	0.05 (0.40)
	3.0	2.42 (0.57)	1.00 (0.47)	1.88 (0.53)	0.08 (0.43)

*Note:* $c_{1}$ represents the size of stage 1 heterogeneous treatment effects and $c_{2}$ represents the size of stage 2 heterogeneous treatment effects.

To specify heterogeneous treatment effects at both stages, we set ${\tilde{X}}_{20,i}^{T}\psi_{20}=3-Z_{1i}+0.1A_{1i}-0.1Z_{2i}+c_{1}Z_{1i}A_{1i}$ and ${\tilde{X}}_{21,i}^{T}\psi_{21}=-6-4Z_{1i}+5A_{1i}-0.2Z_{2i}$ . Note that $c_{1}$ controls the size and direction of stage 1 heterogeneous treatment effects. To apply the methods, the stage 2 model was specified as $E(Y_{i}|Z_{1i},A_{1i},Z_{2i},A_{2i})={\tilde{X}}_{2i}^{T}\beta_{20}+A_{2i}{\tilde{X}}_{2i}^{T}\beta_{21}$ , where ${\tilde{X}}_{2i}^{T}=(1,Z_{1i},A_{1i},Z_{2i})$ , and the stage 1 model was specified as $E(Y_{i}|Z_{1i},A_{1i},A_{2i}={\hat{d}}_{2i}^{\text{opt}})={\tilde{X}}_{1i}^{T}\beta_{10}+A_{1i}{\tilde{X}}_{1i}^{T}\beta_{11}$ , where ${\tilde{X}}_{1i}^{T}=(1,Z_{1i})$ .

**Figure 2.**
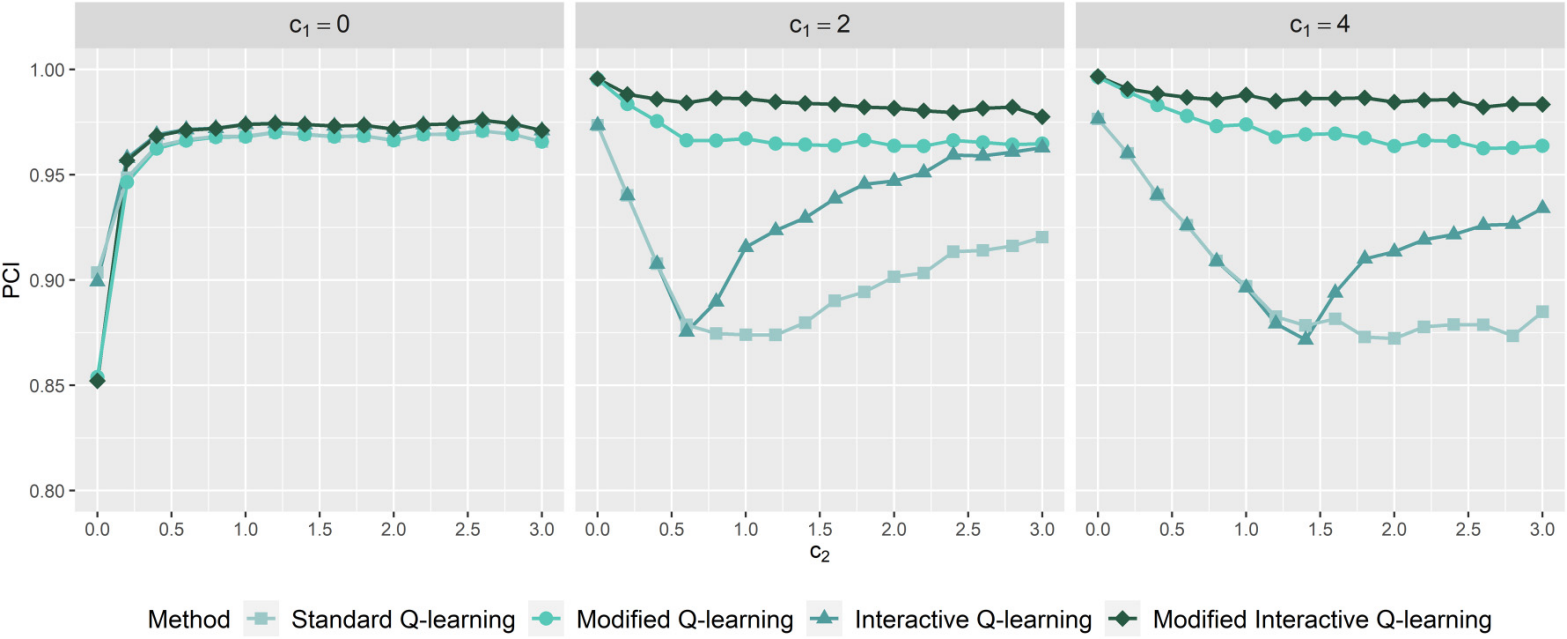
Probability of correctly identified stage 1 optimal rules as a function of $c_{2}$ for $c_{1}=0,2,4$ using standard Q-learning (Q), modified Q-learning (mQ), interactive Q-learning (IQ), and the proposed method (mIQ), based on a set of the population data ( N=10,000 ) and 1000 simulations of the sample data ( n=250 ). Note: $c_{1}$ represents the size of stage 1 heterogeneous treatment effects and $c_{2}$ represents the size of stage 2 heterogeneous treatment effects.

We considered three values of $c_{1}=0,2,4$ . Table 1 summarizes the bias of the estimated optimal value at $c_{2}=1,2,3$ using the four methods considered, and mIQ has the lowest bias across all scenarios. Figure 2 plots the trend in the PCI of stage 1 optimal rules as $c_{2}$ varying from 0 to 3 with an equal interval of 0.2 for each of the three values of $c_{1}$ . If stage 1 treatment effects are homogeneous ( $c_{1}=0$ ), then the interactive algorithms perform slightly better as the stage 2 main effect model is correctly specified so that bias only manifests via the wrongly specified linearity, and mIQ performs exactly the same as IQ. If stage 1 treatment effects are heterogeneous ( $c_{1}\neq 0$ ), then mIQ outperforms all other methods because the modified aspect corrects for the bias of omitting stage 1 heterogeneous treatment effects. As $c_{2}$ increases, a more substantial difference in the PCI of stage 1 optimal rules between mQ and mIQ manifests, which further stresses the importance of building interactive models into modified Q-learning. In summary, the results show that mIQ corrects for potential bias generated from both stage 1 and stage 2 heterogeneous treatment effects.

## 6. Application

We used the M-bridge data ( n=591 ) to illustrate the application of the proposed method on SMARTs with embedded tailoring. Our aim was to identify the personalized optimal DTR that minimizes binge drinking (primary) and negative drinking-related consequences (secondary), respectively, for each subject in the dataset. For the primary outcome, 490 subjects with complete data on the maximum number of drinks within a 24-hour period at baseline and follow-up were included in the analysis. For the secondary outcome, 496 subjects with complete data on the total number of drinking-related consequences in the past 30 days at baseline and follow-up were included in the analysis.

The stage 2 model adjusted for baseline characteristics, including race, gender, intention to pledge Greek, whether a parent had a significant drinking problem, pre-college norms and intention for college drinking, and pre-college drinking habits, as well as their interactions with stage 2 intervention. The stage 1 model included the same baseline characteristics as well as their interaction with stage 1 intervention. The embedded tailoring variable “heavy drinker” determined the subset of re-randomized students which should be included in the stage 2 analysis. A summary of the covariates and outcomes used in the model is presented in Table 2. Among the 591 participants (219 males and 372 females), 11% intended to pledge Greek after attending college, and 13% indicated that a parent had a significant drinking problem. Pre-college drinking norms, intention, and habits were assessed using several metrics via questionnaires. At the end of stage 1 intervention, 158 participants were flagged as heavy drinkers.

**Table 2.** Summary statistics of subject characteristics by initial randomization (stage 1 intervention). Discrete variables are summarized by counts (%); continuous variables are summarized by mean (SD).

	Early intervention	Late intervention	Overall
	( $n_{1}=295$ )	( $n_{2}=296$ )	(n=591)
**Demographics**			
Gender			
Male	115 (39%)	104 (35%)	219 (37%)
Female	180 (61%)	192 (65%)	372 (63%)
Race			
White	229 (78%)	222 (75%)	451 (76%)
Non-White	66 (22%)	74 (25%)	140 (24%)
Intention to pledge Greek			
Yes	34 (12%)	33 (11%)	67 (11%)
No or undecided	261 (88%)	263 (89%)	524 (89%)
Parent drinking problem ${}^{a}$			
Clearly yes	46 (16%)	31 (10%)	77 (13%)
Clearly no or not sure	247 (84%)	264 (89%)	511 (86%)
**Pre-college drinking norms**			
Percent of students drinking ${}^{b}$	53.0 (19.9)	52.4 (21.1)	52.7 (20.5)
Number of drinks per week ${}^{c}$	5.65 (8.35)	5.24 (6.01)	5.45 (7.27)
Max number of drinks in a row ${}^{d}$	5.62 (3.32)	5.35 (3.55)	5.48 (3.44)
Percent of students binge drinking ${}^{e}$	22.4 (17.5)	22.9 (17.8)	22.7 (17.7)
**Intention for college drinking**			
Drinking frequency per month ${}^{f}$	2.37 (2.70)	2.29 (2.80)	2.33 (2.75)
Number of drinks ${}^{g}$	1.98 (1.77)	2.14 (1.91)	2.06 (1.84)
Drunk frequency per month ${}^{h}$	1.39 (1.98)	1.43 (2.28)	1.41 (2.13)
**Pre-college drinking habits**			
Number of days ${}^{i}$	2.05 (3.09)	2.03 (3.25)	2.04 (3.17)
Average number of drinks ${}^{j}$	1.83 (2.37)	1.75 (2.35)	1.79 (2.36)
**Re-randomization/stage 2**			
Heavy drinker	75 (25%)	83 (28%)	158 (27%)
Non-heavy drinker	220 (75%)	213 (72%)	433 (73%)
Stage 2 intervention			
Continued self-monitoring ${}^{k}$	220 (75%)	213 (72%)	433 (73%)
Online health coach	37 (12%)	43 (14%)	80 (14%)
Automated email	38 (13%)	40 (14%)	78 (13%)

${}^{a}$ Indicator whether the subject’s mother or father has had a significant drinking problem that did or should have led to treatment.

${}^{b}$ Norm on the percentage of UMN first-year students who used alcohol during the last 30 days.

${}^{c}$ Norm on the number of alcoholic drinks a typical UMN first-year student consumed during an average week.

${}^{d}$ Norm on the largest number of drinks a typical college student had in a row during the last two weeks.

${}^{e}$ Norm on the percentage of UMN first-year students had five or more drinks in a sitting during the last two weeks.

${}^{f}$ Intent frequency of drinking alcohol per month in the next 6 months.

${}^{g}$ Intent number of drinks on a typical occasion.

${}^{h}$ Intent frequency of consuming enough alcohol to feel drunk or intoxicated per month in the next 6 months.

${}^{i}$ Number of days using alcohol during the last 30 days.

${}^{j}$ Number of drinks had on a typical day when drinking alcohol during the last 30 days.

${}^{k}$ Subjects who were identified as non-heavy drinkers were not randomized at stage 2 and continued self-monitoring.

We used complete-case data for model estimation, thus assuming that any missingness in outcome measurements is not informative, i.e. outcomes are missing at random given baseline covariates. Table 3 summarizes outcomes by the assigned DTR. The missing rate of outcomes is around 16%. For the primary outcome (max_drinks), the stage 2 model utilized data on the 140 heavy drinkers with complete data who were flagged based on the frequency of binge and high-intensity drinking during the self-monitoring period at stage 1, whereas the stage 1 model utilized data on the 490 enrolled students with complete data. Implementing mIQ under the normality assumption, 181 (37.0%) subjects would benefit most from receiving late intervention at stage 1 based on their baseline characteristics, and 90 (64.3%) would benefit most from receiving automated email at stage 2 had they received the predicted stage 1 optimal treatment. For the secondary outcome (byaacq), the stage 2 model utilizes data on the 142 heavy drinkers with complete data, whereas the stage 1 model utilizes data on the 496 enrolled students with complete data. Implementing modified interactive Q-learning, 229 (46.2%) subjects would benefit most from receiving late intervention at stage 1 based on their baseline characteristics, and 77 (54.2%) would benefit most from receiving automated email at stage 2 had they received the predicted stage 1 optimal treatment.

**Table 3.** Summary statistics of outcomes by dynamic treatment regime (DTR). Continuous variables are summarized by mean (SD).

	max_drinks			byaacq		
	n(%)	Baseline	End ${}^{a}$	n(%)	Baseline	End
*All participants*	490	2.55 (3.38)	3.72 (3.68)	496	1.39 (2.42)	2.28 (3.40)
Early	245 (50%)	2.74 (3.37)	3.70 (3.62)	249 (50%)	1.47 (2.48)	2.37 (3.52)
Late	245 (50%)	2.36 (3.37)	3.75 (3.76)	247 (50%)	1.30 (2.36)	2.19 (3.27)
*Heavy drinkers*	140 (29%)	5.26 (3.68)	7.16 (3.21)	142 (29%)	2.99 (2.85)	4.92 (4.24)
Early/email	35 (7%)	5.83 (3.17)	7.20 (3.22)	36 (7%)	2.89 (3.06)	5.06 (4.04)
Early/coach	33 (7%)	4.94 (3.08)	7.15 (2.68)	33 (7%)	2.97 (2.26)	5.18 (4.77)
Late/email	32 (7%)	3.78 (2.66)	6.03 (2.86)	33 (7%)	2.79 (2.46)	4.15 (4.06)
Late/coach	40 (8%)	6.22 (4.81)	8.03 (3.69)	40 (8%)	3.28 (3.43)	5.22 (4.18)

${}^{a}$ End of Semester 1.

We applied and compared all four methods discussed (Q, mQ, IQ, and mIQ) and summarized the analysis results in Table 4. Q-learning is able to make different recommendations to subjects by virtue of treatment effect heterogeneity. Table 4 does not reveal very distinctive recommendations across the methods. For the primary outcome (max_drinks), standard Q-learning and the modified counterpart with Murphy’s regret function generate similar results, indicating that the effect of stage 1 intervention is not substantially heterogeneous. However, there is a slightly more substantial difference in the results between standard and interactive Q-learning, indicating that the heterogeneity of stage 2 intervention effects is relatively stronger. This is verified by Figure D.1(a) in Supplemental Appendix D.1, where the estimated stage 1 heterogeneous treatment effects are distributed around 0 with a very small dispersion. Moreover, residual diagnostics of the parsimonious stage 2 model used in the analysis do not differ much from those of the saturated model including all previous treatment interactions (Supplemental Figure D.2). Therefore, mIQ is anticipated to perform similarly to IQ, which is indeed confirmed by the analysis results. For the secondary outcome (byaacq), the results differ more across the methods and indicate that the intervention effects on drinking-related consequences are slightly more heterogeneous across participants.

**Table 4.** Data analysis results using all four methods: standard Q-learning (Q), modified Q-learning (mQ), interactive Q-learning (IQ), and the proposed method (mIQ).

		Stage 1 prediction			Stage 2 prediction	
Method	$n_{1}$	${\hat{d}}_{1}^{\text{opt}}=-1$	${\hat{d}}_{1}^{\text{opt}}=1$	$n_{2}$	${\hat{d}}_{2}^{\text{opt}}=-1$	${\hat{d}}_{2}^{\text{opt}}=1$
max_drinks
Q	490	219 (44.7%)	271 (55.3%)	140	90 (64.3%)	50 (35.7%)
mQ	490	224 (45.7%)	266 (54.3%)	140	89 (63.6%)	51 (36.4%)
IQ	490	187 (38.2%)	303 (61.8%)	140	90 (64.3%)	50 (35.7%)
mIQ	490	181 (37.0%)	309 (63.0%)	140	90 (64.3%)	50 (35.7%)
byaacq
Q	496	298 (60.1%)	198 (39.9%)	142	78 (54.9%)	64 (45.1%)
mQ	496	261 (52.6%)	235 (47.4%)	142	77 (54.2%)	65 (45.8%)
IQ	496	237 (47.8%)	259 (52.2%)	142	79 (55.6%)	63 (44.4%)
mIQ	496	229 (46.2%)	267 (53.8%)	142	77 (54.2%)	65 (45.8%)

Note that we assumed normality of stage 2 heterogeneous treatment effects for the interactive model building of Q-learning, that is, $g({\tilde{X}}_{21}^{T}\psi_{21}|H_{1},A_{1})$ follows a normal distribution. This assumption can be tested visually or statistically. We plotted residuals against fitted values, standardized residuals against theoretical quantiles (Q–Q plot), and a histogram of residuals (Supplemental Figure D.3). Although the residuals generally display a normal pattern, there are outliers at both tails that deviate from the straight identity line in the Q–Q plot and result in heavy tails in the histogram. We also performed the Shapiro-Wilk test^23^ to avaluate the normality hypothesis. The test is significant ( p<0.0001 ) for both outcomes, indicating that stage 2 heterogeneous treatment effects are not normally distributed. Therefore, we should instead use nonparametric and empirical distribution function^24,25^ to estimate the g function for the purpose of providing more robustness when the normality assumption is violated. The analysis results are summarized in Table 5.

**Table 5.** Data analysis results using interactive model building methods with normal distribution (-normal) and nonparametric (-nonpar) empirical distribution for g .

		Stage 1 prediction			Stage 2 prediction	
Method	$n_{1}$	${\hat{d}}_{1}^{\text{opt}}=-1$	${\hat{d}}_{1}^{\text{opt}}=1$	$n_{2}$	${\hat{d}}_{2}^{\text{opt}}=-1$	${\hat{d}}_{2}^{\text{opt}}=1$
max_drinks
IQ-normal	490	187 (38.2%)	303 (61.8%)	140	90 (64.3%)	50 (35.7%)
IQ-nonpar	490	188 (38.4%)	302 (61.6%)	140	90 (64.3%)	50 (35.7%)
mIQ-normal	490	181 (37.0%)	309 (63.0%)	140	90 (64.3%)	50 (35.7%)
mIQ-nonpar	490	200 (40.8%)	290 (59.2%)	140	90 (64.3%)	50 (35.7%)
byaacq
IQ-normal	496	237 (47.8%)	259 (52.2%)	142	79 (55.6%)	63 (44.4%)
IQ-nonpar	496	213 (42.9%)	283 (57.1%)	142	77 (54.2%)	65 (45.8%)
mIQ-normal	496	229 (46.2%)	267 (53.8%)	142	77 (54.2%)	65 (45.8%)
mIQ-nonpar	496	216 (43.5%)	280 (56.5%)	142	77 (54.2%)	65 (45.8%)

To better describe the treatment effect heterogeneity and understand which variables are contributing to the heterogeneity, we applied a random forest technique^26^ to identify the significant predictors driving different recommendations (more details in Supplemental Appendix C). For the aim of minimizing the primary outcome, the analysis results show that students with the intention and habit of drinking more would benefit more from late intervention at stage 1, and heavy drinkers whose parent had a significant drinking problem would benefit more from online health coach at stage 2. For the aim of minimizing the secondary outcome, in contrast, non-white students may benefit more from late intervention at stage 1, and heavy drinkers who intended to pledge Greek would benefit more from automated email at stage 2.

## 7. Conclusion

In this article, we proposed a modified interactive Q-learning algorithm to attenuate the impact of model misspecification as a result of heterogeneous treatment effects at multiple stages. A major contribution we make to the literature is the attempt to understand and quantify the part of the bias caused by misspecified main effect model of the stage 2 Q-function, specifically, omitting an unmeasured variable, and we confirmed the existence of bias when the unmeasured variable correlates with the treatment at stage 1. To address this bias, we modified the Q-learning algorithm with Murphy’s regret function to account for the unexplained residuals. This modification was then built into interactive Q-learning, which corrects the bias generated by stage 2 heterogeneous treatment effects and the optimization operation, to improve the overall performance where both stage 1 and 2 treatment effects are believed to be heterogeneous. In the conventional practice of Q-learning, stage 1 heterogeneous treatment effects might be a significant predictor at stage 1 but are usually overlooked in the stage 2 main effect model. By reasoning the bias associated with heterogeneous treatment effects and developing the proposed solution, we would like to draw the attention of clinical investigators and policy makers to the high possibility of model misspecification in analyzing data collected from a SMART with possible treatment effect moderators. We do not claim to “eliminate” the bias of standard Q-learning because the impact of heterogeneous treatment effects might not be exhaustively explored in the sequentially randomized design. Rather, we attenuate the bias by addressing the two known sources simultaneously. Future work on identifying other sources of bias due to the interplay of heterogeneous treatment effect and multiple stages of treatment would be valuable.

Omission of unmeasured variables in the main effect model is an intrinsic problem in Q-learning, especially for high-dimensional moderators or SMARTs with more than two stages, where it is impractical to include all important higher-order interactions with treatment from previous stages in the current Q-function. A significant advantage of our proposed method is that, for model building at a specific stage, there is no need to assume all earlier-stage treatment effects are correctly captured and included in the main effect model at the current stage, which is unverifiable due to the backward nature of Q-learning. In practice, existence of high-dimensional covariates may be computationally problematic and we can apply regularization or dimension reduction techniques (e.g. principal component analysis or variable selection based on random forest) to select important moderators into the treatment effect model at each stage without worrying about the main effect model, where any residual bias caused by omitting important interactions from previous stages could be taken care of by our proposed method. In contrast, standard Q-learning requires investigators to include all these important interactions in the main effect model to obtain unbiased estimators.

The trial setting in which our proposed method could be applied is not limited to the M-bridge study design where two treatment arms are considered at each stage. This method is generalizable to comparison among multiple treatment options by rewriting the framework using dummy treatment variables, representing heterogeneous treatment effects using contrasts, and estimating the optimal decision rule by searching through the available treatment options for which treatment minimizes the Q-function at each stage. A difficulty with this generalization, however, is that the treatment component of $Y^{\text{opt}}$ cannot be simply written as the absolute value function of heterogeneous treatment effects so that the integral in Step (mIQ-4) may not have a closed form and nonparametric estimation by empirical distribution should beused.

Both the simulation study and the trial design in our application have equal/balanced allocation of the randomization arms, but this is not required to ensure unbiased estimation of the treatment effects. All that is required is perfect randomization, that is, the “no unmeasured confounders” assumption, and a fixed treatment allocation so that the treatment variable can be specified to have expectation zero. As evidenced by Supplemental Theorem A.2, it is necessary to have $E(A_{2})=0$ as we do not know for sure whether the omitted variable is correlated with the predictors already included in the model (usually an untestable condition in practice). If the randomization scheme has an unbalanced allocation ratio, for example, 2:1 allocation for stage 2 treatments A and B, then we can code the treatment variable as $A_{2}=1$ for treatment A and −2 for treatment B such that $E(A_{2})=0$ . In a more complex trial setting where confounders may be present, one could apply inverse probability of treatment weights to establish balance.

The M-bridge study monitored participant outcomes repeatedly at stage 2, but we only considered the single measurement immediately following stage 2 intervention. Further study of our proposed method in the context of full utilization of the outcome trajectory, that is, analysis of repeated-measures outcomes using generalized estimating equations^27^ instead of linear regression is needed.

## Supplemental Material

sj-pdf-1-smm-10.1177_09622802231206471 - Supplemental material for Modified interactive Q-learning for attenuating the impact of model misspecification with treatment effect heterogeneity Click here for additional data file. Supplemental material, sj-pdf-1-smm-10.1177_09622802231206471 for Modified interactive Q-learning for attenuating the impact of model misspecification with treatment effect heterogeneity by Yuan Zhang, David M Vock, Megan E Patrick and Thomas A Murray in Statistical Methods in Medical Research
