# Supplementary material for: Modified interactive Q-learning for attenuating the impact of model misspecification with treatment effect heterogeneity
Source: Stat Methods Med Res. 2023 Oct 20;32(11):2240–53. doi: 10.1177/09622802231206471 (PMC10683339; doi:10.1177/09622802231206471)
Supplement: sj-pdf-1-smm-10.1177_09622802231206471 - Supplemental material for Modified interactive Q-learning for attenuating the impact of model misspecification with treatment effect heterogeneity [file sj-pdf-1-smm-10.1177_09622802231206471.pdf]

Supplementary Materials for "Modified Interactive Q-learning for Attenuating the Impact of Model Misspecification with Treatment Effect Heterogeneity"

Yuan Zhang[1], David M. Vock[2], Megan E. Patrick[3], and Thomas A. Murray[2]

[1]*Department of Biostatistics, Epidemiology and Informatics, Perelman School of Medicine, University of Pennsylvania, Philadelphia, Pennsylvania, USA*

[2]*Division of Biostatistics, School of Public Health, University of Minnesota, Minneapolis, Minnesota, U.S.A.*

[3]*Institute for Social Research, University of Michigan, Ann Arbor, Michigan, U.S.A.*

Appendix A Unmeasured Variables

A.1 Misspecification of Stage 2 Main Effect Model

We start with model misspecification in the presence of an important variable prior to stage 2 randomization which is not measured by design or omitted in the data analysis. Huang et al. (2015) [1] argues that model misspecification causes bias in the estimation of stage 1 optimal rules and model parameters. We attempt to identify the source of bias and briefly outline the argument here. Let $\boldsymbol{V}_{20}$ denote a vector of unmeasured covariates at stage 2 that are independent of A_2. We use uppercase to denote random variables and lowercase to denote a realization of the corresponding random variable. Suppose Y conditional on all the covariates, measured or unmeasured, has mean

$$\mathbb{E}(Y|H_2, A_2, \boldsymbol{V}_{20}) = \psi_{200} + \boldsymbol{x}_{20}^T\boldsymbol{\psi}_{201} + \boldsymbol{V}_{20}^T\boldsymbol{\gamma}_{20} + a_2\big(\psi_{210} + \boldsymbol{x}_{21}^T\boldsymbol{\psi}_{211}\big) \tag{A.1}$$

and variance $\mathrm{Var}(Y|H_2, A_2, \boldsymbol{V}_{20}) = \sigma_2^2$.

A.2 Estimation Bias

The proof of the theorems can be found in Section B.

Theorem A.1 (Matrix Version of the Omitted Variable Bias Theorem)**.** *Suppose that the true regression model for Y is $Y = \psi_0 + \boldsymbol{X}^T\boldsymbol{\psi}_1 + \boldsymbol{V}^T\boldsymbol{\gamma} + \varepsilon$, where $\boldsymbol{X}$ is a random vector formed by measured covariates, $\boldsymbol{V}$ is formed by unmeasured covariates, and $\varepsilon \sim \mathcal{N}(0, \sigma^2)$. The parameters associated with measured covariates, $\boldsymbol{\psi} \equiv (\psi_0,\, \boldsymbol{\psi}_1^T)^T$, are thus estimated via the misspecified model $y = \beta_0 + \boldsymbol{x}^T\boldsymbol{\beta}_1 + \varepsilon^*$, where y and $\boldsymbol{x}$ are realizations of Y and $\boldsymbol{X}$, respectively. Then*

$$\mathbb{E}(\widehat{\boldsymbol{\beta}}) \equiv \mathbb{E}\begin{pmatrix}\widehat{\beta}_0\\ \widehat{\boldsymbol{\beta}}_1\end{pmatrix} = \boldsymbol{\psi} + \begin{pmatrix}\mathbb{E}(\boldsymbol{V}^T) - \mathbb{E}(\boldsymbol{X}^T)\mathrm{Cov}(\boldsymbol{X})^{-1}\mathrm{Cov}(\boldsymbol{X}, \boldsymbol{V})\\ \mathrm{Cov}(\boldsymbol{X})^{-1}\mathrm{Cov}(\boldsymbol{X}, \boldsymbol{V})\end{pmatrix}\boldsymbol{\gamma}.$$

Let $\boldsymbol{X}_2 = (\boldsymbol{X}_{20}^T,\, A_2,\, A_2\boldsymbol{X}_{21}^T)^T$ denote the full predictor vector and assume that the covariance matrix of $\boldsymbol{X}_2$ is invertible. By Theorem A.1,

$$\mathbb{E}\begin{pmatrix}\widehat{\beta}_{200}\\ \widehat{\boldsymbol{\beta}}_2\end{pmatrix} = \begin{pmatrix}\psi_{200}\\ \boldsymbol{\psi}_2\end{pmatrix} + \mathcal{B}\boldsymbol{\gamma}_{20},$$

where

$$\mathcal{B} = \begin{pmatrix}\mathbb{E}(\boldsymbol{V}_{20}^T) - \mathbb{E}(\boldsymbol{X}_2^T)\mathrm{Cov}(\boldsymbol{X}_2)^{-1}\mathrm{Cov}(\boldsymbol{X}_2, \boldsymbol{V}_{20})\\ \mathrm{Cov}(\boldsymbol{X}_2)^{-1}\mathrm{Cov}(\boldsymbol{X}_2, \boldsymbol{V}_{20})\end{pmatrix}.$$

The first element of $\mathcal{B}$ corresponds to the bias associated with the intercept term and the second element of $\mathcal{B}$ corresponds to the bias associated with the covariate predictors. The existence of bias in the estimation of covariate effects is then characterized by the term $\mathrm{Cov}(\boldsymbol{X}_2, \boldsymbol{V}_{20})$. The bias of $\widehat{\boldsymbol{\beta}}_2$ can be rewritten as

$$\mathrm{Bias}(\widehat{\boldsymbol{\beta}}_2) = \mathrm{Cov}(\boldsymbol{X}_2)^{-1}\begin{pmatrix}\mathrm{Cov}(\boldsymbol{X}_{20}, \boldsymbol{V}_{20})\\ \mathrm{Cov}(A_2, \boldsymbol{V}_{20})\\ \mathbb{E}(A_2)\mathrm{Cov}(\boldsymbol{X}_{21}, \boldsymbol{V}_{20})\end{pmatrix}\gamma_{20},$$

and $\mathrm{Cov}(A_2, \boldsymbol{V}_{20}) = \boldsymbol{0}^T$ for SMARTs due to sequential randomization.

Theorem A.2 (Bias of Stage 2 Treatment Effect Estimators)**.** *Assume that $\boldsymbol{V}_{20}$ is a vector of unmeasured covariates that are independent of A_2 and $\mathrm{Cov}(\boldsymbol{X}_2)$ is invertible. The estimators of stage 2 heterogeneous treatment effects are unbiased if and only if at least one of the following conditions is satisfied:*

- $\mathbb{E}(A_2) = 0$*;*

- $\boldsymbol{V}_{20}$ *is correlated with neither* $\boldsymbol{X}_{20}$ *nor* $\boldsymbol{X}_{21}$*.*

Theorem A.3 (Bias of Stage 2 Main Effect Estimators)**.** *Assume that* $\boldsymbol{V}_{20}$ *is a vector of unmeasured covariates that are independent of* A_2 *and* $\mathbb{E}(A_2) = 0$*. Suppose that* $\boldsymbol{V}_{20}$ *is correlated with* $\boldsymbol{X}_{20}$ *and* $\mathrm{Cov}(\boldsymbol{X}_2)$ *is invertible. Then the estimators of stage 2 main effects are biased and the bias is* $\mathcal{B}'\boldsymbol{\gamma}_{20}$*, where*

$$\mathcal{B}' = \begin{pmatrix} \mathbb{E}(\boldsymbol{V}_{20}^T) - \mathbb{E}(\boldsymbol{X}_2^T)\mathrm{Cov}(\boldsymbol{X}_2)^{-1}\mathrm{Cov}(\boldsymbol{X}_2, \boldsymbol{V}_{20}) \\ \mathrm{Cov}(\boldsymbol{X}_{20})^{-1}\mathrm{Cov}(\boldsymbol{X}_{20}, \boldsymbol{V}_{20}) \end{pmatrix}. \tag{A.2}$$

Theorem A.2 shows the importance of balancing sample size in the randomization arms. With unbalanced designs, it is possible to bias the estimation of stage 2 treatment effects. However, this is not the case we consider in this paper. In the M-bridge study, heavydrinkers were re-randomized to $A_2 = 1$ and $A_2 = -1$ with equal probabilities, i.e., $\mathbb{E}(A_2) = 0$, so no bias would be induced in the identification of stage 2 optimal rules. Theorem A.3 shows that the estimators of stage 2 main effects, however, can be biased if the unmeasured variable $\boldsymbol{V}_{20}$ is correlated with $\boldsymbol{X}_{20}$.

Appendix B Proof

Theorem A.1 (Matrix Version of the Omitted Variable Bias Theorem)**.** *Suppose that the true regression model for* Y *is* $Y = \psi_0 + \boldsymbol{X}^T\boldsymbol{\psi}_1 + \boldsymbol{V}^T\boldsymbol{\gamma} + \varepsilon$*, where* $\boldsymbol{X}$ *is a random vector formed by measured covariates,* $\boldsymbol{V}$ *is formed by unmeasured covariates, and* $\varepsilon \sim \mathcal{N}(0, \sigma^2)$*. The parameters associated with measured covariates,* $\boldsymbol{\psi} \equiv (\psi_0,\, \boldsymbol{\psi}_1^T)^T$*, are thus estimated via the misspecified model* $y = \beta_0 + \boldsymbol{x}^T\boldsymbol{\beta}_1 + \varepsilon^*$*, where* y *and* $\boldsymbol{x}$ *are realizations of* Y *and* $\boldsymbol{X}$*, respectively. Then*

$$\mathbb{E}(\widehat{\boldsymbol{\beta}}) \equiv \mathbb{E}\begin{pmatrix} \widehat{\beta}_0 \\ \widehat{\boldsymbol{\beta}}_1 \end{pmatrix} = \boldsymbol{\psi} + \begin{pmatrix} \mathbb{E}(\boldsymbol{V}^T) - \mathbb{E}(\boldsymbol{X}^T)\mathrm{Cov}(\boldsymbol{X})^{-1}\mathrm{Cov}(\boldsymbol{X}, \boldsymbol{V}) \\ \mathrm{Cov}(\boldsymbol{X})^{-1}\mathrm{Cov}(\boldsymbol{X}, \boldsymbol{V}) \end{pmatrix}\boldsymbol{\gamma}.$$

Theorem A.1 is developed based on the *omitted variable formula* and example in *Econometric Analysis* by Greene (2002) [2].

Proof. Let $\mathbf{X}$ denote a design matrix with $\boldsymbol{x}_i^T$ as the ith row, $i = 1, \ldots, n$ and $\boldsymbol{y}$ denote a vector with y_i as the ith element. Suppose that $\boldsymbol{V}$ is observable and let $\mathbf{V}$ denote a matrix with $\boldsymbol{v}_i$, a realization of $\boldsymbol{V}$, as the ith row. Define $\widetilde{\mathbf{X}} = (\mathbf{1}\ \mathbf{X})$, where $\mathbf{1}$ is a column of 1's. The least squares

estimator of $\boldsymbol{\beta}$ is $\widehat{\boldsymbol{\beta}} = (\widetilde{\mathbf{X}}^T\widetilde{\mathbf{X}})^{-1}\widetilde{\mathbf{X}}^T\boldsymbol{y}$. Thus,

$$\mathbb{E}(\widehat{\boldsymbol{\beta}}) = \boldsymbol{\psi} + \mathbb{E}\left[(\widetilde{\mathbf{X}}^T\widetilde{\mathbf{X}})^{-1}\widetilde{\mathbf{X}}^T\mathbf{V}\right]\boldsymbol{\gamma}.$$

Consider the multivariate regression

$$\boldsymbol{V} = \boldsymbol{\lambda}_0 + \boldsymbol{\Lambda}_1\boldsymbol{x} + \boldsymbol{\varepsilon}^{**}, \text{ where } \boldsymbol{\varepsilon}^{**} \sim \mathcal{MVN}(\mathbf{0}, \Sigma).$$

The least squares estimator of $\boldsymbol{\Lambda} = \begin{pmatrix}\boldsymbol{\lambda}_0^T \\ \boldsymbol{\Lambda}_1^T\end{pmatrix}$ is $\widehat{\boldsymbol{\Lambda}} = (\widetilde{\mathbf{X}}^T\widetilde{\mathbf{X}})^{-1}\widetilde{\mathbf{X}}^T\mathbf{V}$. Hence, $\mathbb{E}\left[(\widetilde{\mathbf{X}}^T\widetilde{\mathbf{X}})^{-1}\widetilde{\mathbf{X}}^T\mathbf{V}\right] = \mathbb{E}(\widehat{\boldsymbol{\Lambda}}) = \boldsymbol{\Lambda}$ and $\mathbb{E}(\widehat{\boldsymbol{\beta}}) = \boldsymbol{\psi} + \boldsymbol{\Lambda}\boldsymbol{\gamma}$.

Now, it remains to prove that $\boldsymbol{\Lambda} = \begin{pmatrix}\mathbb{E}(\boldsymbol{V}^T) - \mathbb{E}(\boldsymbol{X}^T)\mathrm{Cov}(\boldsymbol{X})^{-1}\mathrm{Cov}(\boldsymbol{X}, \boldsymbol{V}) \\ \mathrm{Cov}(\boldsymbol{X})^{-1}\mathrm{Cov}(\boldsymbol{X}, \boldsymbol{V})\end{pmatrix}$. The true values of $\boldsymbol{\Lambda}$ satisfy the equation

$$(\boldsymbol{\lambda}_0, \boldsymbol{\Lambda}_1) = \arg\min_{\boldsymbol{\Lambda}} \mathbb{E}(\boldsymbol{V} - \boldsymbol{\lambda}_0 - \boldsymbol{\Lambda}_1\boldsymbol{X})^T(\boldsymbol{V} - \boldsymbol{\lambda}_0 - \boldsymbol{\Lambda}_1\boldsymbol{X}) = \arg\min_{\boldsymbol{\Lambda}} L.$$

The first derivative of L with respect to $(\boldsymbol{\lambda}_0, \boldsymbol{\Lambda}_1)$ is

$$\begin{aligned}\frac{\partial L}{\partial \boldsymbol{\lambda}_0} &= -2\mathbb{E}(\boldsymbol{V}^T) + 2\boldsymbol{\lambda}_0^T + 2\mathbb{E}(\boldsymbol{X}^T)\boldsymbol{\Lambda}_1^T, \\ \frac{\partial L}{\partial \boldsymbol{\Lambda}_1} &= -2\mathbb{E}(\boldsymbol{X}\boldsymbol{V}^T) + 2\mathbb{E}(\boldsymbol{X})\boldsymbol{\lambda}_0^T + 2\mathbb{E}(\boldsymbol{X}\boldsymbol{X}^T)\boldsymbol{\Lambda}_1^T.\end{aligned}$$

Hence, the score equations of $(\boldsymbol{\lambda}_0, \boldsymbol{\Lambda}_1)$ are

$$\begin{aligned}S(\boldsymbol{\lambda}_0) &= \boldsymbol{\lambda}_0 - \mathbb{E}(\boldsymbol{V}^T) + \mathbb{E}(\boldsymbol{X}^T)\boldsymbol{\Lambda}_1^T = \mathbf{0}, \\ S(\boldsymbol{\Lambda}_1) &= \left[\mathbb{E}(\boldsymbol{X}\boldsymbol{X}^T) - \mathbb{E}(\boldsymbol{X})\mathbb{E}(\boldsymbol{X}^T)\right]\boldsymbol{\Lambda}_1^T - \left[\mathbb{E}(\boldsymbol{X}\boldsymbol{V}^T) - \mathbb{E}(\boldsymbol{X})\mathbb{E}(\boldsymbol{V}^T)\right] \\ &= \mathrm{Cov}(\boldsymbol{X})\boldsymbol{\Lambda}_1^T - \mathrm{Cov}(\boldsymbol{X}, \boldsymbol{V}) = \mathbf{0}\end{aligned}$$

Therefore, $\boldsymbol{\Lambda}_1^T = \mathrm{Cov}(\boldsymbol{X})^{-1}\mathrm{Cov}(\boldsymbol{X}, \boldsymbol{V})$ and $\boldsymbol{\lambda}_0^T = \mathbb{E}(\boldsymbol{V}^T) - \mathbb{E}(\boldsymbol{X}^T)\mathrm{Cov}(\boldsymbol{X})^{-1}\mathrm{Cov}(\boldsymbol{X}, \boldsymbol{V})$. □

Theorem A.2 (Bias of Stage 2 Treatment Effect Estimators)**.** *Assume that $\boldsymbol{V}_{20}$ is a vector of unmeasured covariates that are independent of A_2 and $\mathrm{Cov}(\boldsymbol{X}_2)$ is invertible. The estimators of stage 2 heterogeneous treatment effects are unbiased if and only if at least one of the following conditions is satisfied:*

- $\mathbb{E}(A_2) = 0$;
- $\boldsymbol{V}_{20}$ *is correlated with neither* $\boldsymbol{X}_{20}$ *nor* $\boldsymbol{X}_{21}$.

Proof. By Theorem A.1, we know that the bias of $\widehat{\boldsymbol{\beta}}_2 = \left(\widehat{\boldsymbol{\beta}}_{201}^T, \widehat{\beta}_{210}, \widehat{\boldsymbol{\beta}}_{211}^T\right)^T$ is

$$\text{Bias}(\widehat{\boldsymbol{\beta}}_2) = \text{Cov}(\boldsymbol{X}_2)^{-1}\text{Cov}(\boldsymbol{X}_2, \boldsymbol{V}_{20})\boldsymbol{\gamma}_{20} = \text{Cov}(\boldsymbol{X}_2)^{-1}\begin{pmatrix}\text{Cov}(\boldsymbol{X}_{20}, \boldsymbol{V}_{20})\\ \mathbf{0}^T\\ \mathbb{E}(A_2)\text{Cov}(\boldsymbol{X}_{21}, \boldsymbol{V}_{20})\end{pmatrix}\boldsymbol{\gamma}_{20}.$$

Suppose $\mathbb{E}(A_2) \neq 0$. Since $\text{Cov}(\boldsymbol{X}_2)$ is invertible, for an arbitrary vector $\boldsymbol{\gamma}_{20}$, we have

$$\text{Bias}(\widehat{\boldsymbol{\beta}}_2) = \mathbf{0} \Longleftrightarrow \begin{pmatrix}\text{Cov}(\boldsymbol{X}_{20}, \boldsymbol{V}_{20})\\ \mathbf{0}^T\\ \mathbb{E}(A_2)\text{Cov}(\boldsymbol{X}_{21}, \boldsymbol{V}_{20})\end{pmatrix} = \mathbf{0} \Longleftrightarrow \text{Cov}(\boldsymbol{X}_{20}, \boldsymbol{V}_{20}) = \mathbf{0} \text{ and } \text{Cov}(\boldsymbol{X}_{21}, \boldsymbol{V}_{20}) = \mathbf{0}.$$

Suppose $\mathbb{E}(A_2) = 0$. Note that $\text{Cov}(\boldsymbol{X}_{20}, A_2\boldsymbol{X}_{21}) = \mathbb{E}(A_2)\text{Cov}(\boldsymbol{X}_{20}, \boldsymbol{X}_{21}) = \mathbf{0}$.

Rewrite $\text{Cov}(\boldsymbol{X}_2)$ as a partitioned matrix:

$$\begin{aligned}\text{Cov}(\boldsymbol{X}_2) = \text{Cov}\begin{pmatrix}\boldsymbol{X}_{20}\\ A_2\\ A_2\boldsymbol{X}_{21}\end{pmatrix} &= \begin{pmatrix}\text{Cov}(\boldsymbol{X}_{20}) & \text{Cov}(\boldsymbol{X}_{20}, A_2) & \text{Cov}(\boldsymbol{X}_{20}, A_2\boldsymbol{X}_{21})\\ \text{Cov}(A_2, \boldsymbol{X}_{20}) & \text{Var}(A_2) & \text{Cov}(A_2, A_2\boldsymbol{X}_{21})\\ \text{Cov}(A_2\boldsymbol{X}_{21}, \boldsymbol{X}_{20}) & \text{Cov}(A_2\boldsymbol{X}_{21}, A_2) & \text{Cov}(A_2\boldsymbol{X}_{21})\end{pmatrix}\\ &= \begin{pmatrix}\text{Cov}(\boldsymbol{X}_{20}) & \mathbf{0} & \mathbf{0}\\ \mathbf{0}^T & \text{Var}(A_2) & \text{Var}(A_2)\mathbb{E}(\boldsymbol{X}_{21}^T)\\ \mathbf{0}^T & \text{Var}(A_2)\mathbb{E}(\boldsymbol{X}_{21}) & \text{Cov}(A_2\boldsymbol{X}_{21})\end{pmatrix}.\end{aligned}$$

Let $P = \begin{pmatrix}\text{Var}(A_2) & \text{Var}(A_2)\mathbb{E}(\boldsymbol{X}_{21}^T)\\ \text{Var}(A_2)\mathbb{E}(\boldsymbol{X}_{21}) & \text{Cov}(A_2\boldsymbol{X}_{21})\end{pmatrix}$.

Then $\text{Cov}(\boldsymbol{X}_2)^{-1} = \begin{pmatrix}\text{Cov}(\boldsymbol{X}_{20})^{-1} & \mathbf{0}^T\\ \mathbf{0} & P^{-1}\end{pmatrix}$. It follows that

$$\mathbb{E}(A_2) = 0 \Longleftrightarrow \text{Bias}\begin{pmatrix}\widehat{\beta}_{210}\\ \widehat{\boldsymbol{\beta}}_{211}\end{pmatrix} = P^{-1}\begin{pmatrix}0\\ \mathbb{E}(A_2)\text{Cov}(\boldsymbol{X}_{21}, \boldsymbol{V}_{20})\end{pmatrix} = \mathbf{0}.$$

Therefore, the estimators of stage 2 treatment effects, $\widehat{\beta}_{210}$ and $\widehat{\boldsymbol{\beta}}_{211}$, are unbiased if and only if $\mathbb{E}(A_2) = 0$ or $\text{Cov}(\boldsymbol{X}_{20}, \boldsymbol{V}_{20}) = \text{Cov}(\boldsymbol{X}_{21}, \boldsymbol{V}_{20}) = \mathbf{0}$. □

Theorem A.3 (Bias of Stage 2 Main Effect Estimators)**.** *Assume that* $\boldsymbol{V}_{20}$ *is a vector of unmeasured covariates that are independent of* A_2 *and* $\mathbb{E}(A_2) = 0$*. Suppose that* $\boldsymbol{V}_{20}$ *is correlated with* $\boldsymbol{X}_{20}$ *and* $\text{Cov}(\boldsymbol{X}_2)$ *is invertible. Then the estimators of stage 2 main effects are biased and the bias is* $\mathcal{B}'\boldsymbol{\gamma}_{20}$*, where*

$$\mathcal{B}' = \begin{pmatrix}\mathbb{E}(\boldsymbol{V}_{20}^T) - \mathbb{E}(\boldsymbol{X}_2^T)\text{Cov}(\boldsymbol{X}_2)^{-1}\text{Cov}(\boldsymbol{X}_2, \boldsymbol{V}_{20})\\ \text{Cov}(\boldsymbol{X}_{20})^{-1}\text{Cov}(\boldsymbol{X}_{20}, \boldsymbol{V}_{20})\end{pmatrix}. \tag{A.2}$$

Proof. By Theorem A.1, we know that the bias of $\begin{pmatrix} \widehat{\beta}_{200} \\ \widehat{\boldsymbol{\beta}}_2 \end{pmatrix}$ is

$$\mathcal{B} = \begin{pmatrix} \mathbb{E}(\boldsymbol{V}_{20}^T) - \mathbb{E}(\boldsymbol{X}_2^T)\text{Cov}(\boldsymbol{X}_2)^{-1}\text{Cov}(\boldsymbol{X}_2, \boldsymbol{V}_{20}) \\ \text{Cov}(\boldsymbol{X}_2)^{-1}\text{Cov}(\boldsymbol{X}_2, \boldsymbol{V}_{20}) \end{pmatrix} \gamma_{20}.$$

Note that $\text{Cov}(\boldsymbol{X}_{20}, A_2\boldsymbol{X}_{21}) = \mathbb{E}(A_2)\text{Cov}(\boldsymbol{X}_{20}, \boldsymbol{X}_{21}) = \boldsymbol{0}$. Rewrite $\text{Cov}(\boldsymbol{X}_2)$ as a partitioned matrix:

$$\text{Cov}(\boldsymbol{X}_2) = \text{Cov}\begin{pmatrix} \boldsymbol{X}_{20} \\ A_2 \\ A_2\boldsymbol{X}_{21} \end{pmatrix} = \begin{pmatrix} \text{Cov}(\boldsymbol{X}_{20}) & \text{Cov}(\boldsymbol{X}_{20}, A_2) & \text{Cov}(\boldsymbol{X}_{20}, A_2\boldsymbol{X}_{21}) \\ \text{Cov}(A_2, \boldsymbol{X}_{20}) & \text{Var}(A_2) & \text{Cov}(A_2, A_2\boldsymbol{X}_{21}) \\ \text{Cov}(A_2\boldsymbol{X}_{21}, \boldsymbol{X}_{20}) & \text{Cov}(A_2\boldsymbol{X}_{21}, A_2) & \text{Cov}(A_2\boldsymbol{X}_{21}) \end{pmatrix}$$

$$= \begin{pmatrix} \text{Cov}(\boldsymbol{X}_{20}) & \boldsymbol{0} & \boldsymbol{0} \\ \boldsymbol{0}^T & \text{Var}(A_2) & \text{Var}(A_2)\mathbb{E}(\boldsymbol{X}_{21}^T) \\ \boldsymbol{0}^T & \text{Var}(A_2)\mathbb{E}(\boldsymbol{X}_{21}) & \text{Cov}(A_2\boldsymbol{X}_{21}) \end{pmatrix}.$$

Let $P = \begin{pmatrix} \text{Var}(A_2) & \text{Var}(A_2)\mathbb{E}(\boldsymbol{X}_{21}^T) \\ \text{Var}(A_2)\mathbb{E}(\boldsymbol{X}_{21}) & \text{Cov}(A_2\boldsymbol{X}_{21}) \end{pmatrix}$.

Then $\text{Cov}(\boldsymbol{X}_2)^{-1} = \begin{pmatrix} \text{Cov}(\boldsymbol{X}_{20})^{-1} & \boldsymbol{0}^T \\ \boldsymbol{0} & P^{-1} \end{pmatrix}$ and the bias of $\widehat{\boldsymbol{\beta}}_{201}$ is $\text{Cov}(\boldsymbol{X}_{20})^{-1}\text{Cov}(\boldsymbol{X}_{20}, \boldsymbol{V}_{20})\boldsymbol{\gamma}_{20}$.

□

Appendix C Preliminary Simulation Study

We consider three scenarios for the stage 2 main effects and two scenarios for the stage 2 treatment effects (Table C.1). For the stage 1 treatment effects, we consider two scenarios of homogeneous treatment effects, i.e., the effect of $Z_{1i}A_{1i}$ is 0, and one scenario of heterogeneous treatment effects. The stage 2 treatment effects can be either homogeneous ($\alpha_1 = \alpha_2 = 0$) or heterogeneous ($\alpha_1 = -4, \alpha_2 = -0.2$). The full data generative process is described in Section 5 of the main manuscript.

Table C.1. Specifications of stage 2 main effect and treatment effect model in the data generative mechanism.

**Stage 1 treatment effects** $\widetilde{\boldsymbol{X}}_{20,i}^T\boldsymbol{\psi}_{20} = 3 - Z_{1i} + 0.1A_{1i} - 0.1Z_{2i} + c_1V_i$	**Stage 2 treatment effects** $\widetilde{\boldsymbol{X}}_{21,i}^T\boldsymbol{\psi}_{21} = -6 + \alpha_1 Z_{1i} + 5A_{1i} + \alpha_2 Z_{2i}$
**Homogeneous**	**Homogeneous**
• V_i uncorrelated with H_{2i}: $V_i \sim \mathcal{N}(-1, 1)$	• $\alpha_1 = 0,\ \alpha_2 = 0$
• V_i correlated with H_{2i}: $V_i \sim \mathcal{N}(2Z_{1i}Z_{2i}, 1)$	
**Heterogeneous**	**Heterogeneous**
• $V_i = Z_{1i}A_{1i}$	• $\alpha_1 = -4,\ \alpha_2 = -0.2$

As a preliminary study, we demonstrate existence of the bias caused by an omitted variable in the stage 2 main effect model. The size of the omitted variable can be varied through c_1. We assume that stage 2 treatment effects are homogeneous in this simulation. For each data generative mechanism with one of the three specifications of $\widetilde{\boldsymbol{X}}_{20,i}^T \boldsymbol{\psi}_{20}$, we evaluate the estimators by Monte Carlo integration using samples of size n ($n = 250$ or $n = 2500$) to predict the optimal DTR for a population of $N = 10000$ subjects with known potential outcomes under the four treatment regimes. The stage 2 model was specified as $\mathbb{E}(Y_i|Z_{1i}, A_{1i}, Z_{2i}, A_{2i}) = \widetilde{\boldsymbol{X}}_{2i}^T \boldsymbol{\beta}_{20} + A_{2i}\widetilde{\boldsymbol{X}}_{2i}^T \boldsymbol{\beta}_{21}$, where $\widetilde{\boldsymbol{X}}_{2i}^T = (1, Z_{1i}, A_{1i}, Z_{2i})$ and the stage 1 model was specified as $\mathbb{E}(Y_i|Z_{1i}, A_{1i}, A_{2i} = \widehat{d}_{2i}^{\text{opt}}) = \widetilde{\boldsymbol{X}}_{1i}^T \boldsymbol{\beta}_{10} + A_{1i}\widetilde{\boldsymbol{X}}_{1i}^T \boldsymbol{\beta}_{11}$, where $\widetilde{\boldsymbol{X}}_{1i}^T = (1, Z_{1i})$.

Table C.2. Bias (mean (SD)) of stage 2 main effect estimator $\widetilde{\boldsymbol{X}}_{2i}^T \widehat{\boldsymbol{\beta}}_{20}$ and stage 2 treatment effect estimator $\widetilde{\boldsymbol{X}}_{2i}^T \widehat{\boldsymbol{\beta}}_{21}$ when stage 2 treatment effects are homogeneous across patients ($\alpha_1 = \alpha_2 = 0$) based on a set of test data ($N = 10000$) and 100 simulations of training data.

Omitted Variable	n	$c_1 = 0$	$c_1 = 1$	$c_1 = 2$	$c_1 = 3$	$c_1 = 4$
			Bias$\big(\widetilde{\boldsymbol{X}}_{2i}^T \widehat{\boldsymbol{\beta}}_{20}\big)$			
$V_i \sim \mathcal{N}(-1, 1)$	250	0.01 (0.09)	0.00 (0.14)	0.07 (0.22)	-0.01 (0.28)	0.01 (0.39)
	2500	0.00 (0.03)	0.00 (0.04)	0.02 (0.06)	0.01 (0.09)	-0.03 (0.12)
$V_i \sim \mathcal{N}(2Z_{1i}Z_{2i}, 1)$	250	0.02 (0.08)	0.03 (0.49)	-0.14 (0.86)	-0.22 (1.19)	-0.35 (1.96)
	2500	0.00 (0.03)	0.03 (0.15)	-0.06 (0.31)	0.10 (0.45)	0.26 (0.56)
$V_i = Z_{1i}A_{1i}$	250	0.00 (0.10)	0.03 (0.13)	0.35 (0.21)	2.15 (0.22)	3.15 (0.27)
	2500	0.00 (0.03)	-0.01 (0.04)	0.30 (0.06)	2.06 (0.07)	3.18 (0.08)
			Bias$\big(\widetilde{\boldsymbol{X}}_{2i}^T \widehat{\boldsymbol{\beta}}_{21}\big)$			
$V_i \sim \mathcal{N}(-1, 1)$	250	0.00 (0.09)	0.02 (0.13)	-0.01 (0.23)	0.03 (0.32)	-0.04 (0.37)
	2500	0.00 (0.03)	0.00 (0.05)	0.01 (0.06)	0.02 (0.08)	0.00 (0.12)
$V_i \sim \mathcal{N}(2Z_{1i}Z_{2i}, 1)$	250	-0.01 (0.09)	0.02 (0.47)	0.20 (0.93)	0.01 (1.28)	-0.21 (1.83)
	2500	0.00 (0.03)	0.01 (0.13)	-0.03 (0.29)	-0.03 (0.41)	-0.08 (0.57)
$V_i = Z_{1i}A_{1i}$	250	0.00 (0.09)	0.00 (0.12)	0.03 (0.21)	-0.05 (0.22)	0.03 (0.28)
	2500	0.00 (0.03)	0.00 (0.04)	0.00 (0.06)	0.00 (0.06)	0.00 (0.09)

We summarize the preliminary results in Table C.2 and Table C.3. Table C.2 shows that omission of the stage 1 heterogeneous treatment effects $V_i = Z_{1i}A_{1i}$ in the stage 2 main effect results in a nonnegligible prediction bias of $\widetilde{\boldsymbol{X}}_{2i}^T \widehat{\boldsymbol{\beta}}_{20}$. The stage 2 treatment effects are unbiasedly estimated, shown by the empirically reduced prediction bias with increased sample size, which is as expected from Theorem A.2 as we have a balanced design. Table C.3 shows that omission of $V_i = Z_{1i}A_{1i}$ in the stage 2 main effect model causes significant prediction bias in the stage 1 rule identification. Omission of a variable that is uncorrelated with other stage 2 main predictors does

not cause bias, but if the omitted variable is correlated with other main predictors, then a small bias is generated. Interactive Q-learning indeed does not tackle this problem, but interactive Q-learning has its virtue in correcting the bias caused by falsely assumed stage 1 linear model under heterogeneous stage 2 treatment effects.

Table C.3. Percentage of correctly identified stage 1 optimal rules when stage 2 treatment effects are homogeneous across patients ($\alpha_1 = \alpha_2 = 0$), prediction using standard Q-learning and interactive Q-learning, based on a set of test data ($N = 10000$) and 100 simulations of training data ($n = 250$).

Method	Omitted Variable	$c_1 = 0$	$c_1 = 1$	$c_1 = 2$	$c_1 = 3$	$c_1 = 4$
Standard Q-learning	$V_i \sim \mathcal{N}(-1, 1)$	1	1	1	1	1
	$V_i \sim \mathcal{N}(2Z_{1i}Z_{2i}, 1)$	1	1	0.999	0.996	0.986
	$V_i = Z_{1i}A_{1i}$	1	1	0.965	0.701	0.534
Interactive Q-learning	$V_i \sim \mathcal{N}(-1, 1)$	1	1	1	1	1
	$V_i \sim \mathcal{N}(2Z_{1i}Z_{2i}, 1)$	1	1	0.999	0.992	0.981
	$V_i = Z_{1i}A_{1i}$	1	1	0.965	0.701	0.535

Appendix D Supplementary Materials for Data Analysis

D.1 Supporting Figures

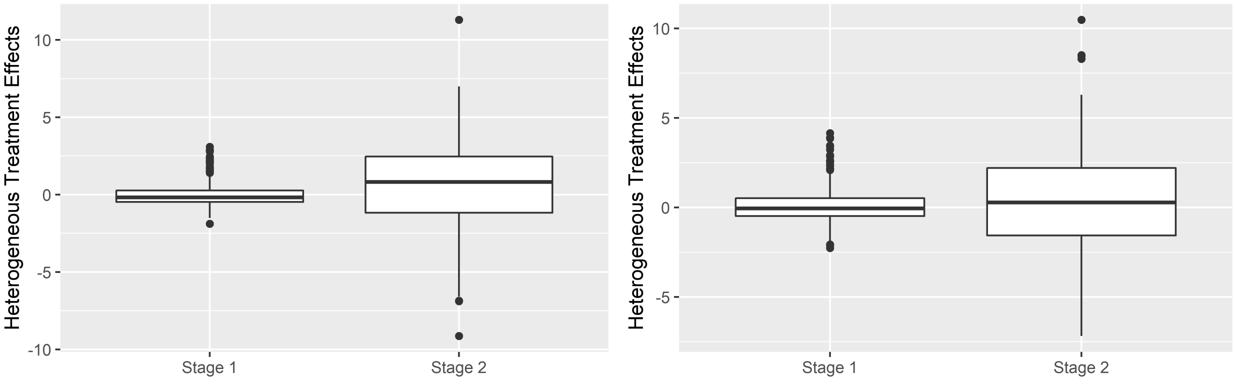

(a) `max_drinks` (b) `byaacq`

Figure D.1. Boxplot of heterogeneous treatment effects at both stage 1 and stage 2 for the outcomes (a) the maximum number of drinks (`max_drinks`) and (b) negative drinking-related consequences (`byaacq`).

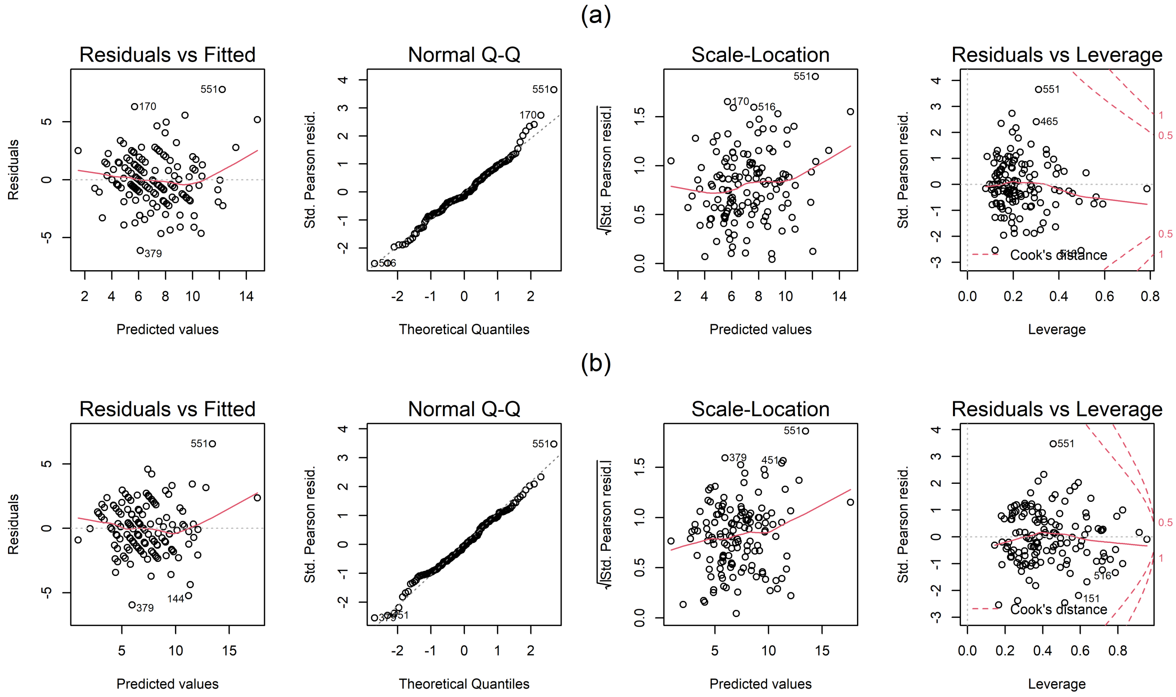

Figure D.2. Residual diagnostics for (a) the parsimonious model (b) the saturated model: an illustration using the primary outcome `max_drinks`.

(a)

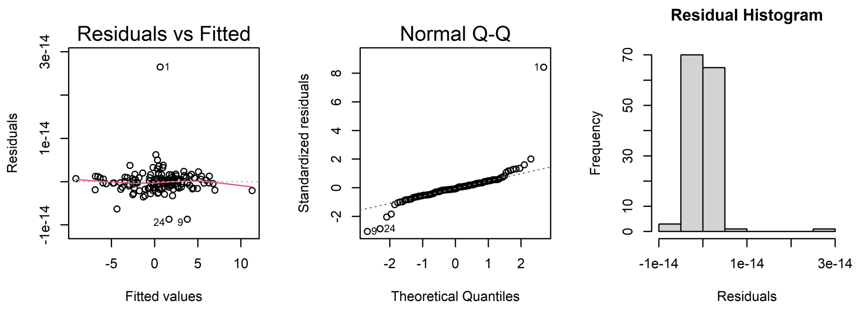

(b)

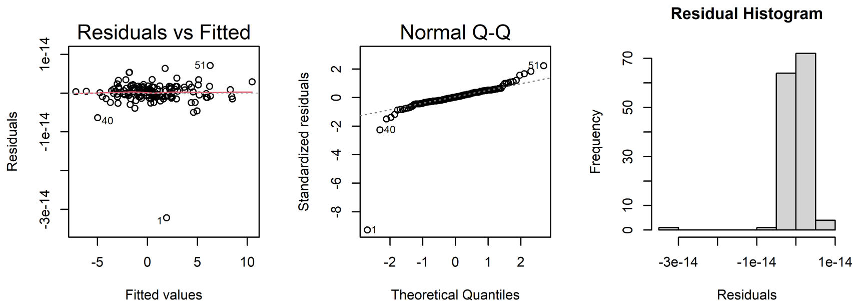

Figure D.3. Model diagnostics for (a) d_1^{opt} and d_2^{opt} and (b) heterogeneous treatment effects at stage 1 and 2, based on the secondary outcome (total number of drinking-related consequences in the past 30 days).

D.2 Identification of Significant Predictors

We determine the most important variables to predict the optimal rules and heterogeneous treatment effects using random forest. The optimal rules are calculated based on an absolute value function of the heterogeneous treatment effects, so their relationship with the predictors is hardly linear and is better represented by decision trees.

Figure D.4 is the variable importance plot for the primary outcome, in which (a) shows the significant variables for predicting the optimal rules and (b) shows the significant variables for predicting the heterogeneous treatment effects, ranked according to their contribution to accuracy and precision. (a) and (b) identify a similar set of significant predictors: at stage 1, the norm on the percentage of first-year college students who had binge drinking during the last two weeks and the habit on the number of days drinking in the past month are the identified variables, whereas at stage 2, whether a student's parent had a significant drinking problem and the intent number of drinks to consume at college on a typical occasion are the identified variables.

(a)

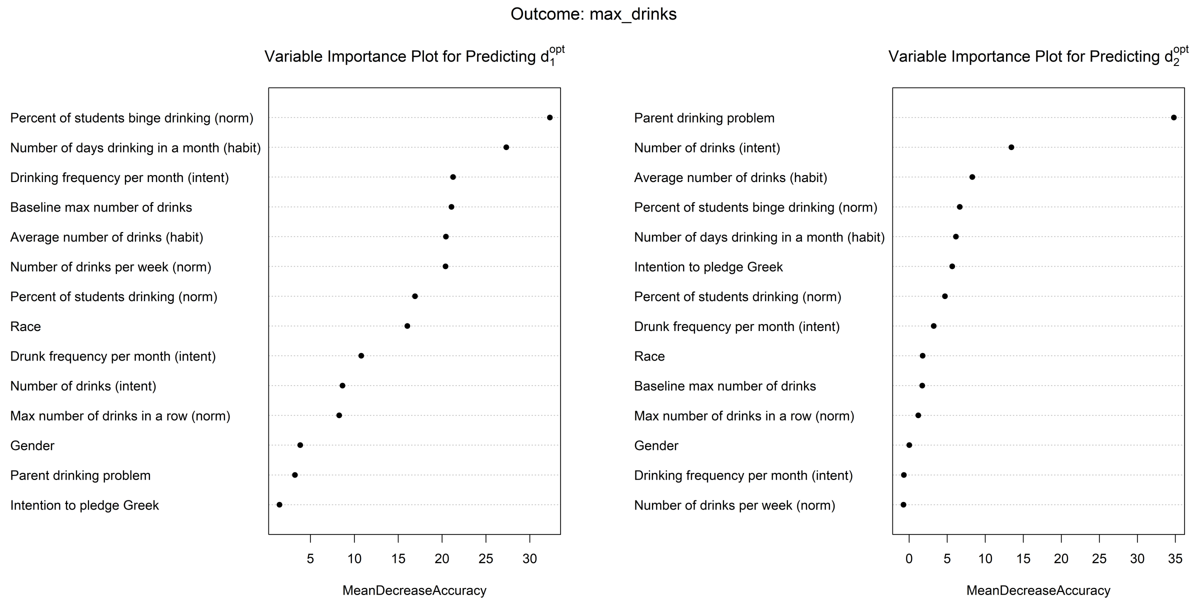

(b)

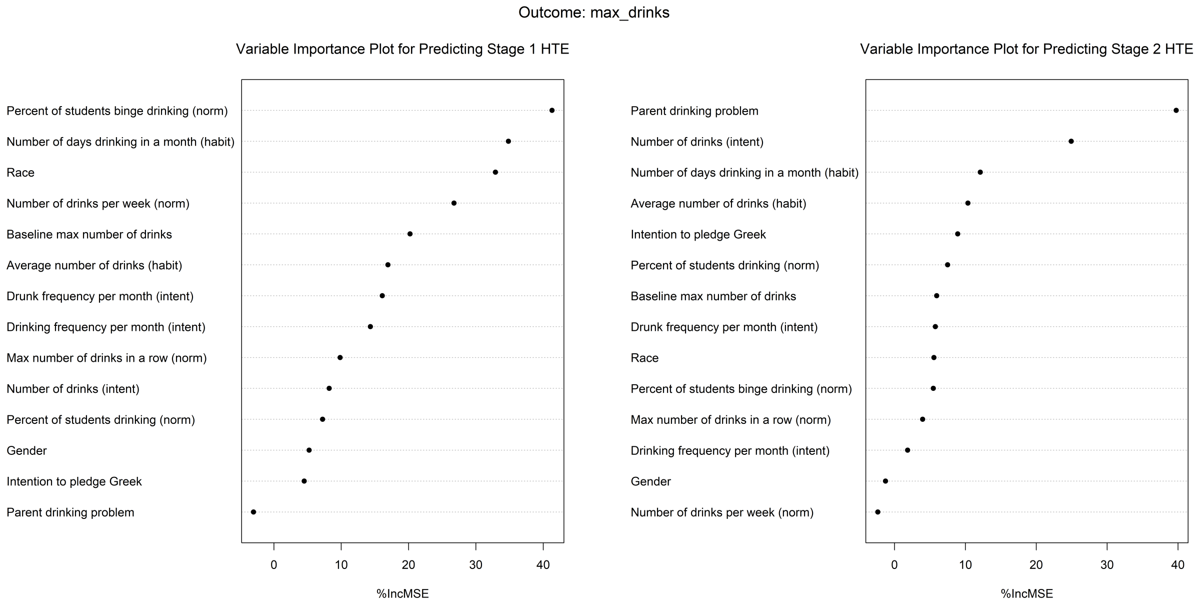

Figure D.4. Variable importance plot for predicting (a) d_1^{opt} and d_2^{opt} and (b) heterogeneous treatment effects at stage 1 and 2, based on the primary outcome (maximum number of drinks consumed within a 24-hour period).

(a)

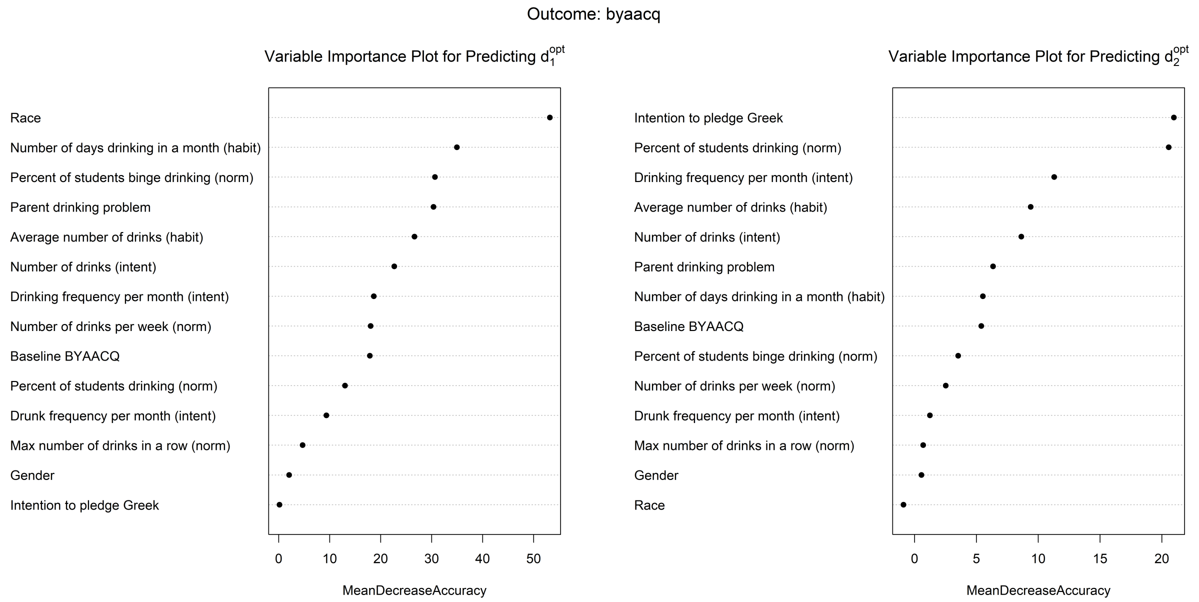

(b)

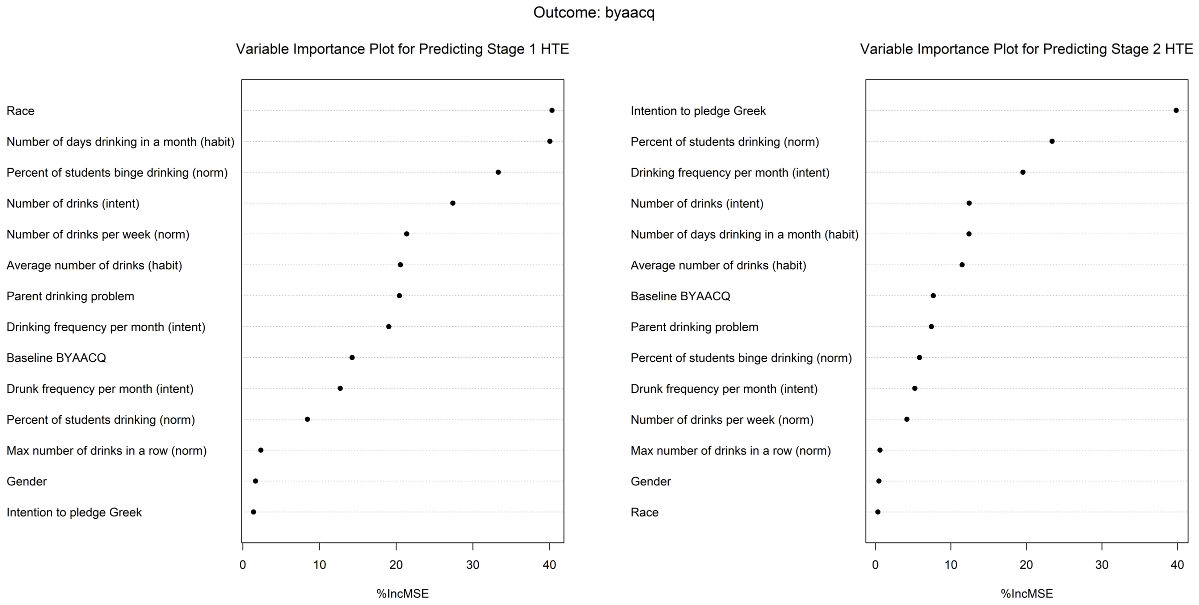

Figure D.5. Variable importance plot for predicting (a) d_1^{opt} and d_2^{opt} and (b) heterogeneous treatment effects at stage 1 and 2, based on the secondary outcome (total number of drinking-related consequences in the past 30 days).

Figure D.5 is the variable importance plot for the secondary outcome. (a) and (b) identify the same set of significant predictors: at stage 1, race, the habit on the number of days drinking in the past month, and the norm on the percentage of first-year college students who had binge drinking during the last two weeks are the identified variables, whereas at stage 2, the intention to pledge to a Greek life, the norm on the percentage of first-year college students who used alcohol in a month, and the intent drinking frequency per month are the identified variables.

Figures D.6 and D.7 show the partial dependence plots for predicting (a) $d_1^{\text{opt}} = 1$ and (b) $d_2^{\text{opt}} = 1$, based on the primary and secondary outcomes, respectively. Each panel in the figure represents a predictor, and the odds that students would benefit more from early intervention at stage 1 or that heavy drinkers would benefit more from online health coach at stage 2 are plotted against the domain of that predictor, so that characteristics associated with high or lower odds can be easily identified. For example, to minimize the maximum number of drinks consumed in a day, students with the intention and habit of drinking more would benefit more from late intervention at stage 1, and heavy drinkers whose parent has a significant drinking problem would benefit more from online health coach at stage 2. Partial dependence plot for predicting $d_2^{\text{opt}} = 1$ is more interpretable and meaningful for investigators to identify the subgroup of students in need of the more expensive intervention (online health coach), and allocate resource correctly. The results reviewed by partial dependence plot for predicting $d_1^{\text{opt}} = 1$ are more intuitive, as completing personalized normative feedback corrects some misperceptions that students with the intention and habit of drinking more would have regarding college drinking, and such students who received the intervention late would have a fresher memory to achieve a more optimal final outcome.

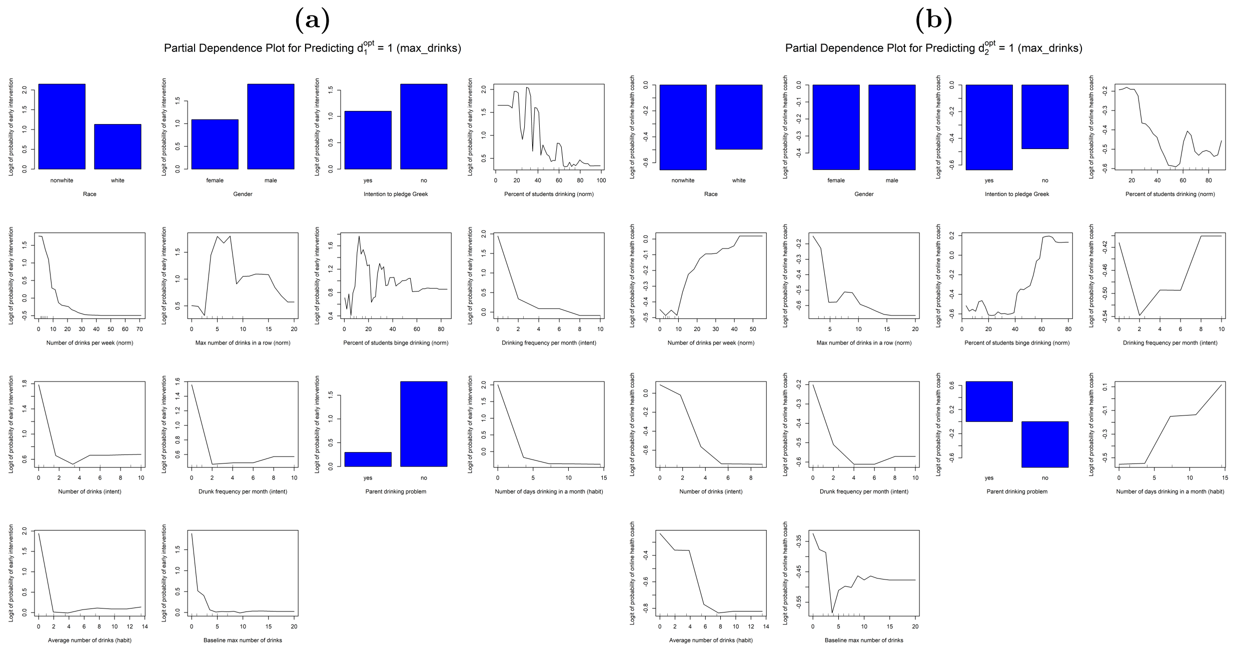

Figure D.6. Partial dependence plot for predicting (a) $d_1^{\text{opt}} = 1$ and (b) $d_2^{\text{opt}} = 1$, based on the primary outcome (maximum number of drinks consumed within a 24-hour period).

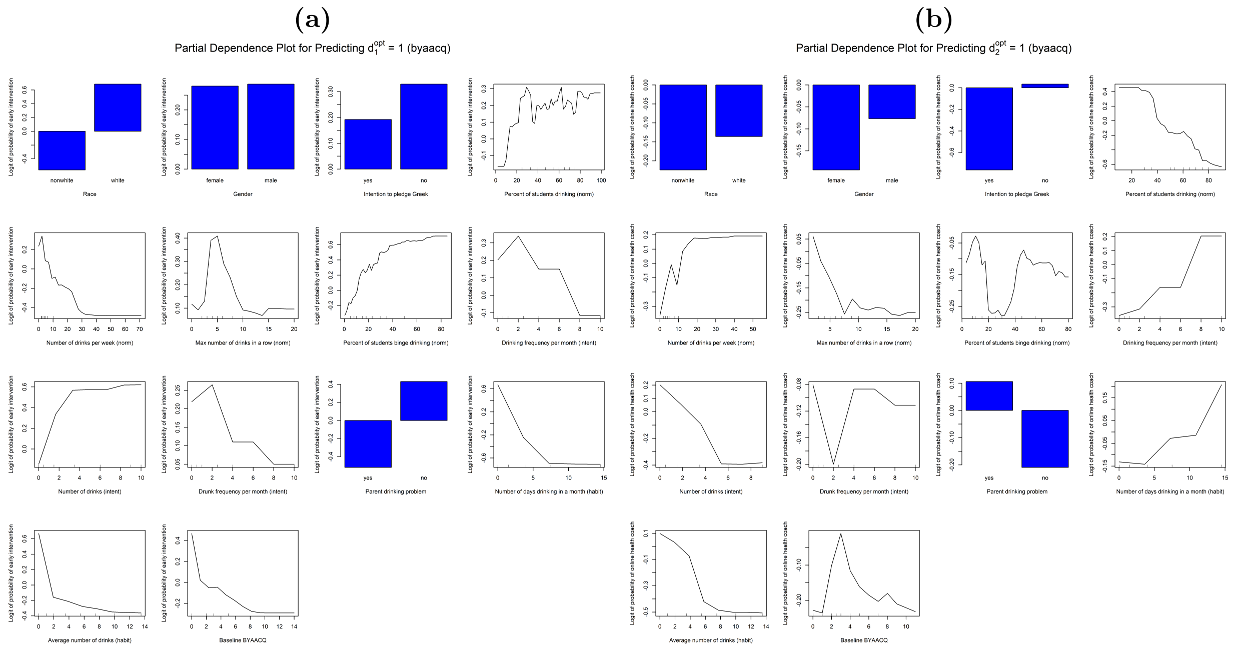

Figure D.7. Partial dependence plot for predicting (a) $d_1^{\text{opt}} = 1$ and (b) $d_2^{\text{opt}} = 1$, based on the secondary outcome (total number of drinking-related consequences in the past 30 days).

References

[1] Huang X, Choi S, Wang L et al. Optimization of multi-stage dynamic treatment regimes utilizing accumulated data. *Statistics in Medicine* 2015; 34(26): 3424–3443.

[2] Greene WH. Specification analysis and model selection. In *Econometric Analysis*. Upper Saddle River, NJ: Prentice Hall, 2002. pp. 148–161.
